# Rewiring carbon metabolism in yeast for high level production of aromatic chemicals

**DOI:** 10.1038/s41467-019-12961-5

**Published:** 2019-10-31

**Authors:** Quanli Liu, Tao Yu, Xiaowei Li, Yu Chen, Kate Campbell, Jens Nielsen, Yun Chen

**Affiliations:** 1 Department of Biology and Biological Engineering, Chalmers University of Technology, Kemivägen 10, SE41296 Gothenburg, Sweden; 2 Novo Nordisk Foundation Center for Biosustainability, Chalmers University of Technology, SE41296 Gothenburg, Sweden; 3 Novo Nordisk Foundation Center for Biosustainability, Technical University of Denmark, DK2800 Kongens Lyngby, Denmark

**Keywords:** Metabolic engineering, Metabolic engineering, Synthetic biology, Applied microbiology

## Abstract

The production of bioactive plant compounds using microbial hosts is considered a safe, cost-competitive and scalable approach to their production. However, microbial production of some compounds like aromatic amino acid (AAA)-derived chemicals, remains an outstanding metabolic engineering challenge. Here we present the construction of a *Saccharomyces cerevisiae* platform strain able to produce high levels of *p*-coumaric acid, an AAA-derived precursor for many commercially valuable chemicals. This is achieved through engineering the AAA biosynthesis pathway, introducing a phosphoketalose-based pathway to divert glycolytic flux towards erythrose 4-phosphate formation, and optimizing carbon distribution between glycolysis and the AAA biosynthesis pathway by replacing the promoters of several important genes at key nodes between these two pathways. This results in a maximum *p*-coumaric acid titer of 12.5 g L^−1^ and a maximum yield on glucose of 154.9 mg g^−1^.

## Abstract (web summary)

Microbial production of aromatic amino acid (AAA)-derived chemicals remains an outstanding metabolic engineering challenge. Here, the authors engineer baker’s yeast for high levels *p*-coumaric acid production by rewiring the central carbon metabolism and channeling more flux to the AAA biosynthetic pathway.

## Introduction

Plant natural products, such as flavonoids and alkaloids, are widely used as food and feed additives, dietary supplements, nutraceuticals, and pharmaceutical drugs^1–4^. However, access to natural sources of these plant-based natural products can often be limited due to their scarce availability. Furthermore, complete chemical synthesis of these compounds has remained challenging due to their structural complexity^5^. To sustainably supply such functional natural products and meet both current and future expected demands, attention has been drawn toward pursuing alternative approaches for their production. Recently, an increasing number of studies have shown that it is possible to reconstitute complex plant pathways in microorganisms for specific plant natural products, such as medicinal opioids, alkaloids, and the precursor to Taxol^6–8^. Subsequently, the feasibility of microbial production for these valuable compounds is increasingly being realized.

The aromatic amino acid (AAA) biosynthesis pathway is one of the core metabolic pathways that lead to the production of many of these specialty compounds. AAAs act as the primary substrates for the biosynthesis of a wide range of commercially relevant natural products, such as flavonoids and alkaloids, which collectively represent a multi-billion-dollar market value. Indeed, there have been several reports on engineering the bacterium *Escherichia coli* for the production of aromatic compounds^9–11^. However, although production in *E. coli* has led to success in some cases, the budding yeast *Saccharomyces cerevisiae* proves to be a more attractive host, due to its robustness and tolerance toward harsh fermentation conditions, and importantly its superior capability of expressing membrane-bound cytochrome P450 oxidases, which are key catalysts in most relevant plant-based biosynthetic pathways^12,13^. Significant efforts have been made toward enhancing the production of AAA-derived products in yeast. Here, the most successful strategies include overexpression of the feedback-insensitive mutant enzymes 3-deoxy-d-arabino-heptulosonate-7-phosphate (DAHP) synthase and chorismate mutase, which catalyze the first committed step in the AAA biosynthesis pathway, and the branching point toward the production of tyrosine and phenylalanine, respectively^14^. However, the reported yields of aromatic compounds in these reports remain too low to make these metabolic-engineering strategies economically feasible for scaling up to industrial levels. In contrast, straightforward genetic modifications such as alleviating feedback inhibition in *E. coli* has resulted in gram-per-liter levels of AAAs and their related products. It is clear therefore that AAA-based production in yeast is currently an outstanding metabolic-engineering challenge.

The initiation of AAA biosynthesis requires two precursors: erythrose-4-phosphate (E4P) derived from the pentose phosphate pathway (PPP) and phosphoenolpyruvate (PEP) derived from glycolysis. Considering the intrinsic functional differences between these parallel pathways, the available fluxes of these two precursors differ drastically. Metabolic flux analysis in yeast has shown that the available carbon flux toward E4P is at least one order of magnitude lower than the flux to PEP, even in a strain that is optimized for initial AAA pathway productivity^12^. Moreover, the enzyme in yeast that catalyzes the first committed step to condense PEP and E4P to form DAHP, DAHP synthase Aro3 and Aro4, both display much higher preferences toward the substrate PEP, with their Michaelis constants (*K*_m_) being 4–7-fold lower than that for their other substrate E4P^15,16^. In addition, E4P has been reported to be the primary limiting substrate for AAA biosynthesis in other microorganisms, such as *E. coli*, *Bacillus subtilis*, and *Corynebacterium glutamicum*^17–19^. Taken together, this would suggest that the key to maximizing the production of aromatic chemicals in yeast is to increase the availability of E4P.

To address the possible constraints on the entrance flux into the AAA biosynthetic pathway, the most common strategy employed by previous studies focuses on rewiring the PPP. This includes overexpression of transketolase Tkl1, which showed to have a limited impact due to kinetic constraints favoring the opposite reaction;^12,20–23^ deletion of glucose-6-phosphate dehydrogenase Zwf1^20,22,24^. The latter, however, blocks the oxidative shunt of the PPP, which is the main source of the redox cofactor NADPH^25^ that is required by the shikimate dehydrogenase Aro1 in the AAA biosynthesis pathway, as well as by many enzymes in downstream pathways for the formation of specialty products. In addition, some other targets including transaldolase Tal1 and ribose-5-phosphate ketol-isomerase Rki1 have also been investigated^22,26^. However, none of these strategies were able to efficiently divert carbon flux from glycolysis toward E4P, to provide sufficient levels for biosynthesis of aromatic chemicals.

**Fig. 1:**
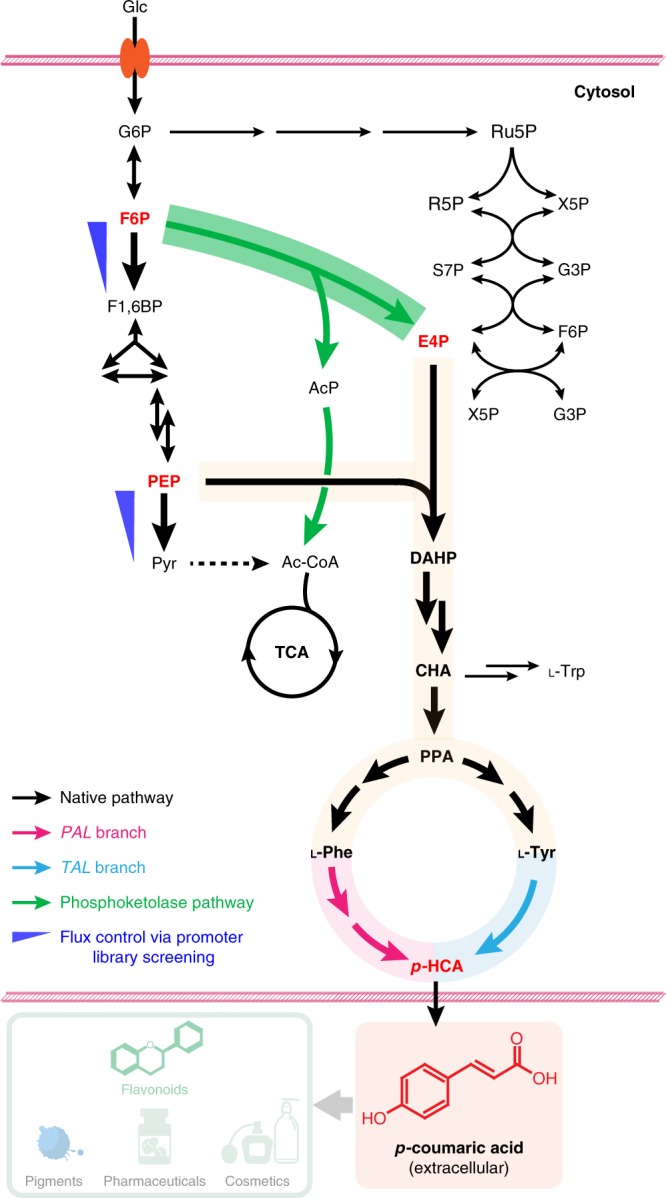
Rewiring yeast central carbon metabolism for efficient production of aromatic chemicals. Introduction of a phosphoketolase (EC 4.1.2.22)-based pathway (green) to divert the glycolytic flux toward the formation of E4P. This consisted of a phosphoketolase to irreversibly cleave F6P into E4P and AcP, and a phosphotransacetylase to convert AcP into acetyl-CoA. The shikimate and AAA biosynthesis pathway were systematically investigated to relieve bottlenecks for biosynthesis of l-Phe and l-Tyr, two substrates for *p*-HCA production. A phenylalanine ammonia lyase and a cinnamic acid hydroxylase (*PAL* branch, pink) and a tyrosine ammonia lyase (*TAL* branch, blue) were both employed for the biosynthesis of *p*-HCA production. Further optimization of carbon distribution between glycolysis and the AAA biosynthesis pathway was achieved by fine-tuning glycolytic flux at phosphofructokinase and pyruvate kinase (blue triangles), via a combinatory promoter screening approach (see the main text). Glc glucose, G6P glucose-6-phosphate, F6P fructose-6-phosphate, F1,6BP fructose 1,6-biphosphate, G3P glyceraldehyde 3-phosphate, PEP phosphoenolpyruvate, Pyr pyruvate, AcP acetyl-phosphate, Ac-CoA acetyl-CoA, TCA tricarboxylic acid cycle, Ru5P ribulose 5-phosphate, R5P ribose-5-phosphate, X5P xylulose-5-phosphate, S7P sedoheptulose 7-phosphate, E4P erythrose-4-phosphate, DAHP 3-deoxy-d-arabino-2-heptulosonic acid 7-phosphate, CHA chorismic acid, PPA prephenate, l-Phe l-phenylalanine, l-Tyr l-tyrosine, l-Trp l-tryptophan, *p*-HCA *p*-coumaric acid

Here we rewire the central carbon metabolism in yeast such that it efficiently provides E4P and channels more flux through the AAA biosynthesis pathway (Fig. 1). In doing so, we can substantially increase the production of AAAs and their derivatives. The contribution of AAA pools toward potential downstream use is evaluated by the formation of *para*-coumaric acid (also called *p*-hydroxycinnamic acid, *p*-HCA), a starting material for a wide array of commercially valuable chemicals from flavors, fragrances, and pharmaceuticals, to biocosmetics, and health and nutrition products.

## Results

### Relieving bottlenecks in the AAA biosynthesis pathway

**Fig. 2:**
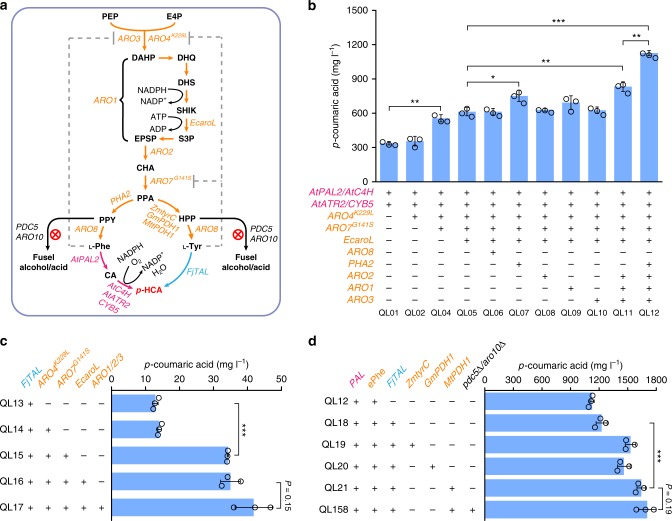
Relieving bottlenecks in the AAA biosynthesis pathway. **a** Overview of yeast metabolic pathway for *p*-HCA biosynthesis. Both the *PAL* branch (pink) consisting of *Arabidopsis thaliana* phenylalanine ammonia lyase (*AtPAL2*), cinnamic acid hydroxylase (*AtC4H*), P450 reductase (*AtATR2*), and yeast native cytochrome b5 (*CYB5*) and the *TAL* branch (blue) consisting of tyrosine ammonia lyase (*FjTAL*) from *Flavobacterium johnsoniae* were constructed for *p*-HCA production. Overexpressed yeast endogenous genes are shown in orange, including DAHP synthase (*ARO3*), l-tyrosine-feedback-insensitive DAHP synthase (*ARO4*^*K229L*^), pentafunctional aromatic protein (*ARO1*), chorismate synthase (*ARO2*), l-tyrosine-feedback-insensitive chorismate mutase (*ARO7*^*G141S*^), prephenate dehydratase (*PHA2*), and aromatic aminotransferase I (*ARO8*). In addition, the *E. coli* shikimate kinase (*EcaroL*) was expressed, and three heterologous l-tyrosine prephenate dehydrogenase encoding genes (*ZmtyrC* from *Zymomonas mobilis*, *GmPDH1* from *Glycine max*, and *MtPDH1* from *Medicago truncatula*) were evaluated for enhancing l-tyrosine supply. The dashed lines indicate feedback inhibition of Aro4 and Aro7 by l-tyrosine and feedback inhibition of Aro3 by l-phenylalanine. Removal of the AAA degradation pathway was enabled by deleting the corresponding genes *PDC5* and *ARO10* (marked with red cross) in the engineered strains. DHQ 3-dehydroquinate, DHS 3-dehydro-shikimate, S3P shikimate-3-phosphate, SHIK shikimate, EPSP 5-enolpyruvyl-shikimate-3-phosphate, PPY phenylpyruvate, HPP *para*-hydroxy-phenylpyruvate, CA cinnamic acid; see Fig. 1 legend regarding abbreviations of other metabolites. *p*-HCA titers obtained with engineered strains derived from the *PAL* branch **b**, the *TAL* branch **c**, and the combination of both branches and the removal of the AAA degradation pathway **d**, respectively. ePhe represents the combination of identified upstream beneficial manipulations (shown in Fig. 2b) for phenylalanine biosynthesis. Cells were grown in defined minimal medium with six tablets of FeedBeads as the sole carbon source, and cultures were sampled after 96 h of growth for *p*-HCA detection. Statistical analysis was performed by using Student’s *t* test (one-tailed; two-sample unequal variance; *p < 0.05, **p < 0.01, ***p < 0.001). All data represent the mean of *n* = 3 biologically independent samples and error bars show standard deviation. The source data underlying figures **b**–**d** are provided in a Source Data file

The production of *p*-HCA in yeast can be accomplished via a single heterologous enzymatic step from tyrosine (the *TAL* branch) or via two enzymatic steps from phenylalanine (the *PAL* branch) (Figs. 1 and 2a). By introducing (i) a phenylalanine ammonia lyase (*AtPAL2*), a cinnamic acid hydroxylase (*AtC4H*), and a cytochrome P450 reductase (*AtATR2*) from *Arabidopsis thaliana*, together with a cytochrome B5 (*CYB5*) from *S. cerevisiae*, or (ii) a highly specific tyrosine ammonia lyase from *Flavobacterium johnsoniae* (*FjTAL*)^27^, we generated the reference strains QL01 and QL13 that were able to produce *p*-HCA with titers of 337.6 and 12.9 mg L^−1^, respectively (Fig. 2b, c, Supplementary Fig. 1 for more strain information). Clearly, the *PAL* branch was far more efficient compared with the *TAL* branch, despite intracellular tyrosine concentrations usually reported as being slightly higher than those of phenylalanine during glucose-limited conditions^28,29^, which is similar to our slow release of glucose conditions used here. Our results could also be attributed to the low efficiency of the tyrosine ammonia lyase used here. Expressing feedback-insensitive DAHP synthase Aro4^K229L^ in either of the two reference strains did not significantly improve *p*-HCA production; however, accumulation of AAA biosynthesis pathway intermediates such as shikimate, and by-products such as tryptophol, phenylethanol, and *p*-hydroxyphenylethanol, was significantly increased in strain QL02 compared with QL01 (Supplementary Fig. 2), consistent with the previous report on the alleviation of the feedback allosteric loop on DAHP synthase^14^. In line with this, overexpression of feedback-insensitive chorismate mutase Aro7^G141S^ alone did not have a statistically significant (*p* > 0.05) effect on *p*-HCA production (Supplementary Fig. 3). However, by creating a strain with both Aro4^K229L^ and Aro7^G141S^ modifications, *p*-HCA production increased by about 1.7-fold with the *PAL* branch (strain QL04) and 2.7-fold with the *TAL* branch (strain QL15) (Fig. 2b, c). This indicates the importance of relieving allosteric regulation of DAHP synthase and chorismite mutase to increase carbon flux toward phenylalanine and tyrosine, a finding that was further supported by reducing tryptophol accumulation in strain QL04 (Supplementary Fig. 2). On the other hand, further increase seen in levels of the pathway intermediate shikimate indicated that additional bottlenecks still existed after alleviation of these feedback allosteric loops.

As the heterologous shikimate kinase AroL from *E. coli* has previously been shown to be beneficial for production of AAA-derived chemicals^30^, introduction of *EcaroL* was tested (resulting in strains QL05 and QL16); however, this only showed modest effects on *p*-HCA production. Next, we therefore systematically overexpressed genes in the AAA biosynthesis pathway to identify additional bottlenecks. For this, we used the strain QL05 harboring the *PAL* branch, as it had the highest flux toward *p*-HCA. Among the genes tested, overexpression of *PHA2* (strain QL07, Fig. 2b) led to a significant (*p* < 0.05) increase in *p*-HCA production by about 23%, compared with strain QL05. Though individual expression of *ARO2*, *ARO1*, or *ARO3* did not display any significant effect on the production of *p*-HCA, combining all three genes increased *p*-HCA production by 36% (strain QL11, Fig. 2b) in comparison with strain QL05. Moreover, when introducing Pha2 together with Aro1, 2, and 3 (strain QL12, Fig. 2b), *p*-HCA production almost doubled compared with that of QL05, clearly pointing to there being untapped potential at this point in the metabolic network for enlarging flux through the AAA biosynthesis pathway.

Overexpression of Aro1, 2, and 3 (strain QL17) in the *TAL* branch however, only had a moderate impact on the production of *p*-HCA, compared with strain QL16 (Fig. 2c), suggesting that the availability of tyrosine as a precursor is not limiting for *p*-HCA production. This led us to speculate that tyrosine ammonia lyase may be a bottleneck. Interesting though, when we combined *FjTAL* with the enhanced phenylalanine (ePhe)-*PAL* branch (strain QL12) giving strain QL18, *p*-HCA production increased by a factor of 99 ± 36 mg L^−1^ (Fig. 2d), which was much larger than *p*-HCA production conferred by the *TAL* branch alone (strain QL13, Fig. 2c), pointing to a synergistic effect between these two pathways. It is not clear whether prephenate dehydrogenase Tyr1, which is native to yeast, is regulated by tyrosine concentrations, and if overexpression of this enzyme would have a negative impact on *p*-HCA production^30^. We therefore turned to other variants of Tyr1, such as *PDH1* from *Glycine max* (*GmPDH1*) and *Medicago truncatula* (*MtPDH1*), and *tyrC* from *Zymomonas mobilis* (*ZmtyrC*) to see if this would alleviate possible limitations in metabolic flux, which had been mediated by the native Tyr1. Indeed, all three variants were able to enhance *p*-HCA production, with MtPDH1 showing the highest increase in production, giving a *p*-HCA titer of 1641.7 mg L^−1^ and a yield of 82.1 mg g^−1^ on glucose (strain QL21, Fig. 2d).

After remedying the carbon flux from the precursors PEP and E4P toward *p*-HCA, we next tested the impact of reducing by-product formation in strain QL21 by deletion of *PDC5* and *ARO10*, as has been previously implemented^30,31^. The resulting double-deletion strain (QL158) did not significantly (*p* = 0.19) improve *p*-HCA production (Fig. 2d). However, deletion of *PDC5* and *ARO10* had a significant effect on cell growth (Supplementary Fig. 4), leading us to conclude that these deletions, at least at this stage, were unsuitable for integrating into our engineering strains. To further increase the flux through the AAA biosynthesis pathway, we next moved to increasing the availability of E4P, a limiting precursor for AAA biosynthesis.

### Rewiring carbon distribution toward E4P

**Fig. 3:**
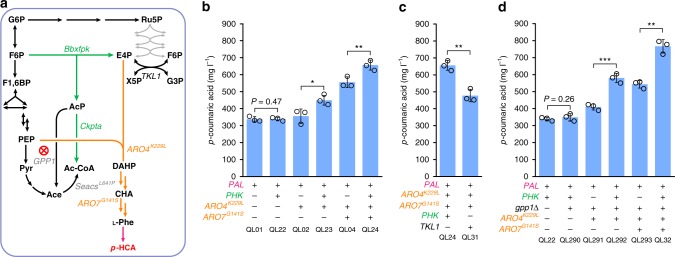
Validation of a phosphoketolase-mediated E4P generation route in the basic *PAL* branch strains. **a** Schematic overview of phosphoketolase (PHK)-based pathway for the generation of E4P for *p*-HCA production. The heterologous PHK pathway, consisting of a phosphoketolase from *Bifidobacterium breve* (BbXfpk) and a phosphotransacetylase from *Clostridium kluyveri* (CkPta), was introduced into the *PAL* branch-based (including a phenylalanine ammonia lyase and a cinnamic acid hydroxylase) strain. Ace acetate; see Fig. 1 legend regarding abbreviations of other metabolites. **b** Introduction of the PHK pathway in combination with feedback-insensitive DAHP synthase (*ARO4*^*K229L*^), and chorismate mutase (*ARO7*^*G141S*^) increases *p*-HCA titers via the *PAL* branch. **c** The PHK pathway outperforms the native E4P-generating route via Tkl1 for *p*-HCA production. **d** Combining the PHK pathway with deletion of the native glycerol-1-phosphatase-encoding gene *GPP1* (marked with a red cross in **a**) enhances *p*-HCA production. Cells were grown in defined minimal medium with six tablets of FeedBeads as the sole carbon source, and cultures were sampled after 96 h of growth for *p*-HCA detection. Statistical analysis was performed by using Student’s *t* test (one-tailed; two-sample unequal variance; *p < 0.05, **p < 0.01, ***p < 0.001). All data represent the mean of *n* = 3 biologically independent samples and error bars show standard deviation. The source data underlying figures **b**–**d** are provided in a Source Data file

To overcome the intrinsic scarcity of E4P required for AAA biosynthesis, we implemented a heterologous phosphoketolase (PHK) pathway, including a phosphoketolase from *Bifidobacterium breve* (*Bbxfpk*) and a phosphotransacetylase from *Clostridium kluyveri* (*Ckpta*). Phosphoketalose is able to split fructose-6-phosphate into E4P and acetyl-phosphate (Fig. 3a); therefore, introduction of this pathway could, theoretically, divert part of the carbon flux from glycolysis directly toward E4P. This theory was indeed supported by intracellular sugar phosphate profiling, showing that E4P concentration was enhanced by 5.4-fold in the BbXfpk-expressing strain^32^. DAHP synthase has low affinity toward E4P (*K*_m_ of 500 µM, Aro4)^16^, and elevated levels of E4P should therefore increase the flux through this first committed step of AAA biosynthesis.

To validate our strategy, we started with a simple background strain that contained only the *PAL* branch pathway for *p*-HCA production. However, insertion of this pathway alone into the basic *PAL* branch strain had no significant impact (*p* = 0.47) on *p*-HCA production (strain QL22, Fig. 3b), even when this was coupled to the overexpression of wild-type DAHP synthase Aro4 (Supplementary Fig. 5). As there is tight regulation on DAHP synthase, we also combined the PHK pathway with a feedback-insensitive DAHP synthase mutant Aro4^K229L^ (strain QL23), enabling a 26% improvement in *p*-HCA production compared with strain QL02 (Fig. 3b). Moreover, combining this pathway with the expression of both Aro4^K229L^ and Aro7^G141S^ (strain QL24) led to the production of 656.4 mg L^−1^ of *p*-HCA, a 18% enhancement in *p*-HCA production levels, compared with strain QL04 (Fig. 3b).

Phosphoketolase expression was also found to have a positive effect on *p*-HCA production in the *TAL* branch (Supplementary Fig. 6). These results suggest that the introduction of the PHK pathway is effective in channeling more carbon flux toward E4P formation, thus enhancing the production of *p*-HCA. As a comparison, we also tested the overexpression of the transketolase-encoding gene *TKL1*, a common strategy implemented in both *E. coli*^17^ and in *S. cerevisiae*^20^ for optimizing E4P formation. Tkl1 overexpression, however, resulted in a lower titer of *p*-HCA production (strain QL31, Fig. 3c) compared with the introduction of the PHK pathway (strain QL24, Fig. 3c), which, maybe not surprisingly, correlates with the preference of Tkl1 for catalyzing the opposite reaction (consuming E4P)^20^.

During shake-flask cultivation, the PHK pathway-containing strain (QL24) exhibited a reduced specific growth rate, from 0.35 to 0.29 h^−1^, and an increased accumulation of acetate, from 0.32 to 1.10 g L^−1^, in comparison with strain QL04, which did not contain this pathway (Supplementary Fig. 7). These phenotypes have been observed before due, to a large extent, to nonspecific native phosphatases that can degrade the product, acetyl-phosphate, resulting in acetate formation that can hinder cell growth^33,34^. To counter against this, we tested three different strategies: (i) deletion of glycerol-1-phosphatase-encoding gene *GPP1* to reduce acetate formation, (ii) expression of a heterologous acetyl-CoA synthetase variant SeAcs^L641P^ from *Samonella enterica* to increase acetate consumption, or (iii) expression of a second copy of the phosphotransacetylase-encoding gene *Ckpta* to reduce acetate accumulation. The best outcome was with *GPP1* deletion, in which acetate accumulation was significantly reduced (strain QL32, Supplementary Fig. 7), alongside a concomitant increase in *p*-HCA levels by 17% (strain QL32, Supplementary Fig. 8). Due to this positive effect, we decided to combine the PHK pathway with the deletion of *GPP1*, then we reexamined its effect on *p*-HCA production through the *PAL* branch base strains (Fig. 3d). With *GPP1* deletion, the introduction of the PHK pathway enabled more than 40% improvement in *p*-HCA production, demonstrating the potential of this pathway for channeling more flux toward E4P generation (Fig. 3b, d).

### Dynamic control strategy to increase *p*-HCA production

**Fig. 4:**
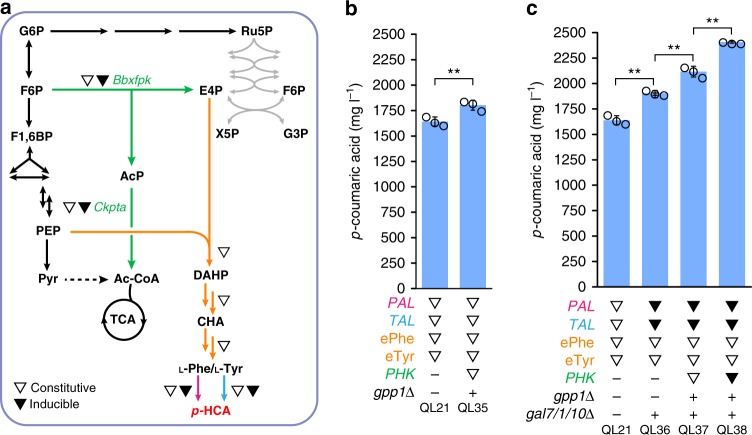
Employment of a combinatorial strategy to increase the production of *p*-HCA. **a** Schematic overview of the metabolic pathway for *p*-HCA production with an improved supply of precursor E4P and dynamic control over the relevant biosynthetic genes, as indicated by triangle symbols: open triangles indicate the use of constitutive strong promoters to control gene expression, while filled triangles indicate the use of galactose-inducible promoters. See Fig. 1 legend regarding abbreviations of metabolites and Fig. 3 legend for gene details. **b** Integration of the PHK pathway with combined ePhe-*PAL* and eTyr-*TAL* branches leads to increased *p*-HCA production. eTyr refers to enhanced tyrosine biosynthesis mediated through the beneficial effect of *MtPDH1*. **c** Dynamic control of biosynthetic genes via use of the *GALp*-controlled expression system significantly increases *p*-HCA production. Cells were grown in defined minimal medium with six tablets of FeedBeads as the sole carbon source and 1% galactose as the inducer when required. Cultures were sampled after 96 h of growth for *p*-HCA detection. Statistical analysis was performed by using Student’s *t* test (one-tailed; two-sample unequal variance; *p < 0.05, **p < 0.01, ***p < 0.001). All data represent the mean of *n* = 3 biologically independent samples and error bars show standard deviation. The source data of figures **b** and **c** are provided in a Source Data file

So far, we have proven that introduction of the PHK pathway is an effective approach to increase the metabolic flux of AAA biosynthesis and subsequently elevate levels of *p*-HCA being produced. We next combined this strategy with an enhanced supply of phenylalanine and tyrosine (ePhe and eTyr, via alleviation of bottlenecks in the AAA biosynthesis pathway) for *p*-HCA production (Fig. 4a). Expression of the PHK pathway genes, *Bbxfpk* and *Ckpta*, under the control of the constitutive strong promoters *TDH3p* and *tHXT7p* in strain QL21, together with the *GPP1* deletion resulted in a *p*-HCA titer of 1802.9 mg L^−1^ (strain QL35), representing a 10% increase compared with strain QL21 (Fig. 4b). However, this increase was less pronounced than the *PAL* branch base strains, which demonstrated more than 40% enhancements upon the same introduction of *GPP1* deletion and PHK pathway (strain QL04 and QL32, Fig. 3b, d), suggesting that downstream flux for converting AAA substrates to *p*-HCA was in some way limited. In addition, a significant decrease in cell growth was observed accompanied by the increased *p*-HCA production for strain QL35 (Supplementary Fig. 9). Such impairment in cell growth may indicate that rewiring carbon distribution by phosphoketolase expression leads to an insufficient supply of carbon needed for the generation of biomass.

To fine-tune the expression levels so far attained, we next employed dynamic control by using the well-characterized *GAL*p expression system to control the expression of heterologous genes that divert AAAs toward *p*-HCA synthesis (Fig. 4a). *GAL* expression is only responsive to galactose exposure without glucose repression, which can in turn facilitate cell growth under excess glucose conditions. The expression of genes under *GAL* promoter control has also shown to be much stronger when compared with normally employed strong constitutive promoters such as *TEF1p* and *TDH3p*^35^. Therefore, the *GALp*-controlled expression system was first tested for both the *PAL* and *TAL* branch genes. To enable this, the structural genes *GAL7*/*1*/*10* were deleted in QL21 to confer galactose as a gratuitous inducer, with *GAL1*, *GAL2*, and *GAL7* promoters being used to control expression of *AtPHA2, AtC4H*, and *FjTAL*. The resultant strain QL36 produced 10% higher of *p*-HCA than that of QL21 (Fig. 4c), which indeed proved that the *GAL* promoters were more suitable in this instance than the constitutive *TEF1* and *TDH3* promoters. Consequently, we then examined phosphoketolase expression in QL36 either under the control of a constitutive promoter or galactose-regulated promoter systems. Strain QL37 with the constitutive promoter control system produced *p*-HCA at a level of 2116.7 mg L^−1^, whereas incorporation of the galactose-induced expression cassette led to a more significant (*p* < 0.01) increase in *p*-HCA titer to 2406.1 mg L^−1^ (strain QL38), a 33% increase compared with QL36 (Fig. 4c). Moreover, when using this background (strain QL36), inducible expression of phosphoketolase showed less significant effects on cell growth (Supplementary Fig. 10).

### Balancing the availability of PEP and E4P

**Fig. 5:**
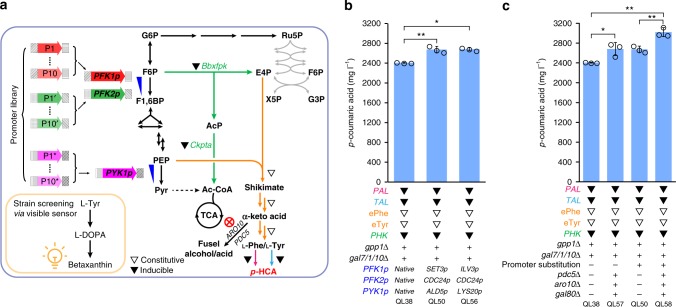
Optimization of carbon distribution increases *p*-HCA production. **a** Schematic illustration of carbon redistribution between glycolysis and the AAA biosynthesis pathway through a promoter library screening approach. A promoter library was created to replace the original promoters of *PFK1*, *PFK2*, and *PYK1*, which encode phosphofructokinase and pyruvate kinase, respectively, at key nodes between glycolysis and the AAA biosynthesis pathway. This promoter library was transformed into a yeast strain harboring the PHK pathway alongside the upregulated shikimate pathway, with the resulting strain screened by using a l-Tyr-derived pathway that indicated increased tyrosine production via the formation of the yellow pigment betaxanthin. Selected promoters exhibiting enhanced color intensity are listed in Supplementary Table 2. Furthermore, AAA degradation pathway was eliminated by deleting the corresponding genes (marked with a red cross) in the final strains. Open triangles indicate the use of constitutive strong promoters for controlling gene expression, while filled triangles indicate the use of galactose-inducible promoters. **b** Simultaneous optimization of the promoters of *PFK1*, *PFK2*, and *PYK1* improves *p*-HCA production. **c** Removing the AAA degradation pathway further enhances *p*-HCA titers. Deletion of *GAL80* was additionally introduced to enable the induction of *GAL* promoters without the addition of galactose. For shake-flask cultivation, cells were grown in defined minimal medium with six tablets of FeedBeads as the sole carbon source and 1% galactose as the inducer when required. For strains with the deletion of *GAL80*, no galactose was supplemented. Cultures were sampled after 96 h of growth for *p*-HCA detection. Statistical analysis was performed by using Student’s *t* test (one-tailed; two-sample unequal variance; *p < 0.05, **p < 0.01, ***p < 0.001). All data represent the mean of *n* = 3 biologically independent samples and error bars show standard deviation. The source data underlying figures **b** and **c** are provided in a Source Data file

Carbon redistribution via the expression of the PHK pathway combined with further optimization by using a *GAL* control system (strain QL38) together, led to about a 50% improvement in *p*-HCA production compared with strain QL21 (Fig. 4c). Next, we asked if the carbon distribution can be further optimized between glycolysis and the AAA biosynthesis pathway through channeling more flux through the PHK pathway. We speculated that one target could be phosphofructokinase, which competes with phosphoketolase for the substrate fructose-6-phosphate (Fig. 5a). We also hypothesized that pyruvate kinase could be another potential target as this enzyme converts most of PEP, the other AAA biosynthesis precursor, into pyruvate (Fig. 5a). To target the controlled expression of these two enzymes, we created a promoter library to replace the original promoters of *PFK1*, *PFK2*, and *PYK1*, which encode phosphofructokinase and pyruvate kinase, respectively. As shown in Fig. 5a, for each of three genes, 10 different promoters, exhibiting differential transcriptional strength in the presence of glucose (based on a previous report^36^), were selected. These promoters were then used to build a promoter library for fine-tuning the expression of *PFK1*/*2* or *PYK1* (a full list of promoters is available in Supplementary Table 1). To screen the promoter-replaced strains in a high-throughput manner, a recently reported l-Tyr-derived pigment, betaxanthin-formation pathway^37^, was employed, allowing carbon redirection to occur alongside colorimetric changes in culture appearance due to the formation of betaxanthin, which is yellow pigmented.

For optimizing this method, we built a betaxanthin pathway that contains two enzymatic steps for converting l-Tyr to betaxanthin by using several different background strains, testing the results both on agar plates and in liquid medium (Fig. 5a and Supplementary Fig. 11). To prevent color saturation at the beginning of the screen, due to the already high flux toward l-Tyr, we used strain QL45, which only had the PHK pathway and feedback-insensitive Aro4^K229L^, as the host for promoter library screening. We then co-transformed 30 promoter cassettes and a triple gRNA vector into this strain and selected transformants by using glucose as the sole carbon source. From these transformants, eight colonies displaying enhanced color intensity were selected and subjected to identification of promoter substitutions (Supplementary Table 2). These strains were then characterized for cell growth and betaxanthin color intensity (Supplementary Fig. 12). Of these promoter substitutions, the two most optimal combinations were as follows: first, the replacement of *PFK1*, *PFK2*, and *PYK1* native promoters with *SET3p*, *CDC24p*, and *ALD5p*; second, the substitution of *PFK1*, *PFK2*, and *PYK1* native promoters with *ILV3p*, *CDC24p*, and *LYS20p*, respectively. Reconstruction of these genes with these promoter replacements in the high producer strain QL38 resulted in strains QL50 and QL56, containing the first and second combination of replacement promoters, respectively (Fig. 5b). Both strains rendered a further increase in the titer of *p*-HCA with the best-performing strain, QL50, producing 2680.2 mg L^−1^ of *p*-HCA, a 11% increase compared with QL38.

Although deletion of the *GAL7*/*1*/*10* gene cluster strains were not able to metabolize galactose, these strains still require a low concentration of galactose for induction of the *PAL*, *TAL*, and PHK pathway genes transcribed by *GAL* promoters. To avoid the use of galactose, we deleted the *GAL80* gene (Fig. 5c), allowing for the induction of galactose-regulated genes upon the release of carbon repression such as by glucose. This approach has been reported to work under similar conditions to ours under glucose-limited fed-batch fermentation conditions without the addition of galactose^38^.

As previously reported, increased fluxes in the AAA biosynthesis pathway led to a significant increase in fusel aromatic compounds^14^ with the deletion of *ARO10* and *PDC5* effectively blocking this route for AAA degradations^30,31^. Indeed, phenylethanol, *p*-hydroxyphenylethanol, and the related acids were found to be accumulated in strain QL38, while knocking out both *ARO10* and *PDC5* (Fig. 5a) significantly reduced these by-products (strain QL57, Supplementary Fig. 13). Interestingly, unlike our earlier attempt to remove the AAA degradation pathway, the introduction of these two deletions at this stage of our work, led to less significant negative impact on cell growth (Supplementary Fig. 14). Consequently, the double deletion was able to further enhance the production of *p*-HCA by 11% (strain QL57, Fig. 5c). These effects were similarly seen in the strain with promoter replacements (QL50). Interestingly, the accumulation of by-products, such as phenylethanol and phenylacetate, was further increased in strain QL50, compared with that detected in QL38 (Supplementary Fig. 13), indicating that the carbon flux through the AAA biosynthesis pathway had been further increased in QL50. As a result, the double deletions in the promoter-substituted strain led to further enhanced *p*-HCA production, reaching a titer of 3021.2 mg L^−1^ in the shake-flask cultivation (strain QL58, Fig. 5c), a 26% improvement compared with QL38. Furthermore, in strain QL58, a yield of 151.1 mg g^−1^ glucose was achieved, corresponding to ~43% of the maximum theoretical yield possible (this being 0.3494 g/g).

### High-level production of *p*-HCA

**Fig. 6:**
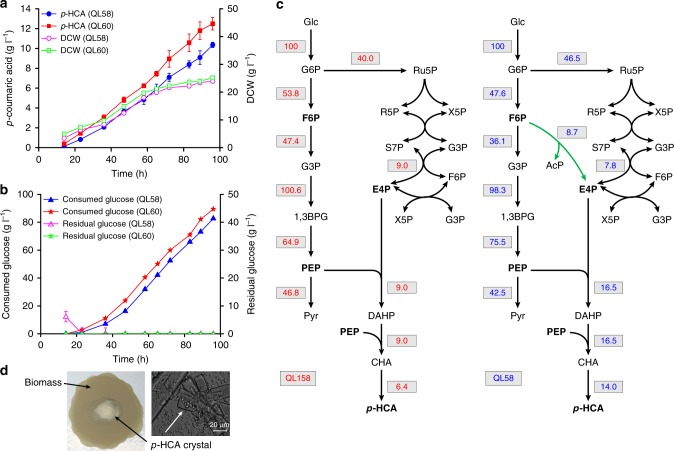
High-level production of *p*-HCA. **a** Fed-batch fermentation of strain QL58 and the corresponding diploid strain QL60 under glucose-limited conditions over time. *p*-HCA titer (filled symbols) and cell mass (open symbols) are shown for each strain. **b** Glucose consumption profile (filled symbols) and time course of residual glucose (open symbols) during the same fed-batch fermentation for each strain. All data represent the mean of *n* = 2 biologically independent samples and error bars show standard deviation. **c** Predicated metabolic flux distributions via flux balance analysis (FBA) in the engineered medium-level *p*-HCA producer QL158 (left, without carbon-rewiring strategy) and high-level *p*-HCA producer QL58 (right, with carbon-rewiring strategy). Based on FBA, the fluxes to the different products were represented relative to the uptake of 100 mmol of glucose. The PHK pathway, the nonnative E4P-forming biosynthetic pathway, is highlighted in green. 1,3BPG 1,3-biphosphoglycerate; see Fig. 1 legend regarding abbreviations of other metabolites. **d** Observed *p*-HCA crystals on filter membrane (left panel) and under a microscope (right panel, scale bar = 20 µm). Crystals of *p*-HCA were formed as a result of its high-level production and relatively low solubility in the fed-batch fermentation broth. The source data underlying figure **a** and **b** are provided in a Source Data file

The production of *p*-HCA in our best-performing strain (QL58) was almost doubled compared with that of QL158, confirming that the PHK pathway-based carbon rewiring strategies we employed could effectively divert more carbon flux toward AAA biosynthesis and *p*-HCA production. In subsequent evaluation of these strains under glucose-limited fed-batch fermentation conditions, QL158 only produced 5.1 g L^−1^ of *p*-HCA, while QL58 produced *p*-HCA at a titer of 10.4 g L^−1^ (Supplementary Fig. 15, Fig. 6a, b). This was consistent with a significant increase (*p* < 0.05) in the rate of *p*-HCA production, which almost doubled in QL58 in comparison with QL158 (Supplementary Table 3). Furthermore, metabolic flux analysis showed that the carbon flux was redistributed among glycolysis, PPP, and AAA biosynthesis pathway in QL58 with the PHK pathway-based carbon-rewiring strategies, compared with that in QL158 lacking these carbon-rewiring strategies (Fig. 6c). These results clearly show that the glycolytic flux was diverted toward E4P formation and AAA biosynthesis, and by redistributing this flux, it was possible to achieve about twofold enhancement of carbon flux toward E4P and AAA biosynthesis in QL58 (Fig. 6c).

As diploid strains typically perform better than the corresponding haploid strains^39^, we further constructed a diploid strain by switching the mating type of QL58, and then mating the resulting strain with QL58 producing a diploid strain with identical chromosome pairs. The resulting diploid strain (QL60) performed slightly better than QL58, with a titer of 3098.7 mg L^−1^ and a yield of 154.9 mg g^−1^ glucose in the shake-flask cultivation (Table 1). Finally, we evaluated the performance of the diploid strain QL60, under glucose-limited fed-batch fermentation conditions, wherein *p*-HCA production reached 12.5 g L^−1^ (Fig. 6a, b and Table 1). In addition, these high titers resulted in crystallization of *p*-HCA in the culture broth (Fig. 6d).

**Table 1:** Comparison of yeast cell factories for production of aromatic chemicals

Target compound	Characteristics of study	Titer (g L^−1^)	Yield (mg g^–1^ glucose)	Productivity (mg L^–1^ h^–1^)	Origin
Shikimic acid	Shake flask	1.979	54.4	ND^a^	^22^
Phenylethanol	Shake flask	0.408	20.4	ND^a^	^57^
Resveratrol	Fed-batch bioreactor	0.812	9.2	7.4	^58^
Naringenin	Batch bioreactor	0.113	5.6	3.0	^31^
Vanillin ß-d-glucoside	Batch bioreactor	0.306	15.3	ND^a^	^59^
Breviscapine	Fed-batch bioreactor	0.293	ND^a^	ND^a^	^60^
*Para*-coumaric acid	Fed-batch deep-well plate	1.93	ND^a^	ND^a^	^30^
*Para*-coumaric acid	Shake flask using FeadBeads^b^	3.02 (3.1)^c^	151.1 (154.9)^c^	31.5 (32.3)^c^	This study
*Para*-coumaric acid	Fed-batch bioreactor	10.4 (12.5)^c^	125.3 (139.6)^c^	107.9 (130.1)^c^	This study

^a^*ND*, not determined

^b^Slow release of glucose from elastomer discs

^c^Values for diploid strains

## Discussion

One of the main bottlenecks in the production of aromatic chemicals occurs in the shikimate and AAA biosynthesis pathway. AAAs are less abundant than other amino acids inside the cell; moreover, the flux toward their biosynthesis is highly regulated. In particular, the availability of E4P is severely limited in many microorganisms^12,17–19^, resulting in a significant constraint on entry flux for AAA biosynthesis. This limitation thus represents a major challenge for producing industrially relevant aromatic chemicals at high levels. In this work, we have successfully rewired yeast central carbon metabolism to enhance flux through the AAA biosynthesis pathway and support biosynthesis of the target molecule, *p*-HCA. This was accomplished by systematically engineering the AAA biosynthesis pathway, introducing a phosphoketalose (EC 4.1.2.22)-based pathway to divert glycolytic flux toward the formation of E4P, and further optimizing the carbon distribution between glycolysis and the AAA biosynthesis pathway through replacing the promoters of several important genes at key nodes between these two pathways, to balance the availability of the AAA precursors PEP and E4P.

Previously, production of *p*-HCA in yeast has been reported at a level of 1.93 g L^−1^, with the process carried out in an intrinsically designed fed-batch complex medium, which releases glucose from higher polysaccharides upon enzyme addition^30^. However, introducing the same modifications only resulted in 22.3 mg L^−1^ of *p*-HCA under our conditions, i.e., normal shake-flask batch cultivation conditions with 2% glucose (Supplementary Fig. 16). This huge difference may well be due to different medium and cultivation conditions used, as we used minimum medium under batch conditions, while Rodriguez et al. used complex medium in fed-batch model. There were also other differences, i.e., components of gene constructs like promoters, terminators, and integration sites, which may also explain the different results obtained. However, the work by Rodriguez et al. did inspire us on the importance of slow glucose release by using, for example, FeedBeads (Kühner AG, Germany), which we found improved the *p*-HCA production without increasing the supply of glucose (Supplementary Fig. 16). This may be due to a higher flux in the PPP under glucose-limited conditions, which in turn can increase the availability of E4P, compared with normal batch conditions^40^. The beneficial effect of the fed-batch model was further confirmed when this strategy was used alongside targeting of the *PAL* branch-based pathway for *p*-HCA production (Supplementary Fig. 17).

Compared with the *TAL* branch, utilization of the *PAL* branch-based pathway demonstrated a significantly improved approach for *p*-HCA production. Moreover, through systematic overexpression of individual genes involved in phenylalanine biosynthesis, Pha2 was identified as a bottleneck in *p*-HCA production (Fig. 2b). Furthermore, this was further proven by overexpression of *PHA2* in a strain with further enhanced flux through the shikimic acid pathway, via additional expression of the PHK pathway together with *GPP1* deleted, leading to a 50% increase in *p*-HCA production (QL288, Supplementary Fig. 18). In contrast to what is known in bacterial systems, no information is available for how prephenate dehydrogenase encoded by *PHA2* is regulated in yeast. These results therefore bring to attention that the formation of phenylpyruvate catalyzed by Pha2 is an important flux-controlling step in the production of phenylalanine and its derivatives.

The synergistic effect of combining the *TAL* branch with the *PAL* branch may well have resulted from relieving feedback inhibition of Tyr1, as the expression of Tyr1 has previously been shown to be inhibited by the presence of phenylalanine^41^. However, by expressing a heterologous variant of Tyr1 from plant *M. truncatula*, we were able to bypass to some extent this feedback inhibition, enabling a further increase in *p*-HCA production by more than 30% (Fig. 2d). These results clearly point to the conversion of prephenate into 4-hydroxyphenylpyruvate, which is catalyzed by Tyr1, to be a limiting step in the production of tyrosine and its derivatives.

After unlocking several bottlenecks in the AAA biosynthesis pathway, we found it to be possible to divert more carbon flux through the shikimate pathway. We were able to enhance the entrance flux toward AAAs and the availability of E4P by expressing a phosphoketolase (EC 4.1.2.22) having high capability for converting fructose-6-phosphate to acetyl-phosphate and E4P^33^. The phosphoketolase enzyme (BbXfpk) used in this study still has a specific activity toward xylulose-5-phosphate, at a greater affinity than that toward fructose-6-phophate, with a ratio close to 3:2. Nonetheless, the intracellular concentrations of fructose-6-phophate under our conditions was present at higher concentrations than xylulose-5-phosphate^42^, making it possible to divert glycolytic flux directly toward the formation of E4P. Indeed, our results showed that expression of this pathway together with feedback-insensitive mutants of Aro4^K229L^ and Aro7^G141S^ improved *p*-HCA production (Fig. 3b). However, one issue with this pathway was that nonspecific native phosphatases, such as Gpp1, could convert the forming product, acetyl-phosphate, into acetate, which showed to have an adverse effect on cell growth^33,34^ (Supplementary Fig. 7). By deleting *GPP1*, we were therefore able to further enhance the production of *p*-HCA levels (Fig. 3d). The production level in the resulting strain QL32 had a 40% improvement when compared with QL04, which only carried the feedback-insensitive mutants of Aro4^K229L^ and Aro7^G141S^ (Fig. 3b, d), clearly demonstrating the potential of this strategy for the production of AAAs and products derived thereof.

The increase in *p*-HCA levels in QL35, through the introduction of the phosphoketolase pathway, was not very significant (10%, Fig. 4b). However, by using dynamic control via the *GALp*-controlled expression system, which fine-tuned phosphoketolase expression as well as the *PAL* and *TAL* branch genes (strain QL38), the production of *p*-HCA could be enhanced by about 50% (Fig. 4c), confirming the power of phosphoketolase to channel carbon flux toward AAA biosynthesis. This could be attributed to two reasons: first, *GALp*-induced expression decoupled cell growth on glucose from the production of *p*-HCA upon galactose induction, which could have in turn enabled the repression of expression during strain construction. This may have thus prevented the deprivation of precursors needed for cell growth or the accumulation of potential toxic intermediates, which could have resulted in strain instability^38^. Second, *GALp* alone has been proven to be an even stronger promoter than those normally employed such as constitutive *TEF1p* and *TDH3p*^35^. On the other hand, these results also implied that channeling AAAs to the production of *p*-HCA could be a limiting factor, as *ARO10* and *PDC5* deletions in QL158 did not redirect carbon flux toward *p*-HCA biosynthesis (Fig. 2d). In line with this, double deletion of *ARO10* and *PDC5* in a strain containing increased upstream carbon flux and optimized downstream flux toward product formation (via dynamic control) led to significantly improved *p*-HCA production (strain QL57, Fig. 5c). In addition, when carbon flux through the AAA biosynthesis pathway had been further increased (QL50, Supplementary Fig. 13), a further increase in *p*-HCA production can be seen with these double deletions (strain QL58, Fig. 5c), which would otherwise have led to production and excretion of fusel aromatic compounds as a carbon sink^14^.

After proving the capability of the PHK pathway to channel carbon flux toward E4P formation and AAA biosynthesis, we next optimized carbon distribution by replacing the promoters for genes *PFK1*/*2* and *PYK1*, two key nodes that can affect the substrate availability for the shikimate pathway (Fig. 5a). By employing a recently published betaxanthin-based screening method, a small library of promoters for these three genes was transformed and selected, to balance the availability of E4P and PEP. Indeed, reconstruction of these promoter substitutions in strain QL38, containing an optimized AAA biosynthesis pathway, resulted in an additional 11% improvement in *p*-HCA production. By combining these promoter replacements with the removal of downstream competing pathways, the production of *p*-HCA could be further improved, reaching a titer of 3021.2 mg L^−1^ in the shake-flask cultivations, and a yield of 151.1 mg g^−1^ on glucose (Fig. 5c). This is an almost twofold increase in total *p*-HCA attained, compared with strain QL158 that lacked this carbon-rewiring strategy.

To discuss and showcase the utility of our optimized strains in the production of other value-added aromatic compounds, we engineered and evaluated the resveratrol biosynthetic pathway in our engineered strains (Supplementary Fig. 19a). Introduction of *Arabidopsis thaliana* 4-coumarate-CoA ligase 1 (*At4CL1*) and *Vitis vinifera* stilbene synthase (*VvSTS*) into strain QL58 produced only 32.1 mg L^−1^ of resveratrol. However, ~2500 mg L^−1^ *p*-HCA accumulated in this strain (QL375, Supplementary Fig. 19b), indicating that another precursor, malonyl-CoA, was limiting. Indeed, by further combination of the deregulated mutant *ACC1*^*S659A, S1157A*^ ^43^, resveratrol production was significantly (*p* < 0.001) increased, reaching a titer of 263.4 mg L^−1^ (QL379, Supplementary Fig. 19b), which is comparable with the level reported for production of this compound in shake-flask cultivation of yeast cells^44^. It is also interesting to be noted that in this strain, almost 2 g L^−1^ *p*-HCA was found to remain, again demonstrating that the strategies we employed were efficient at enhancing carbon flux through the AAA biosynthesis pathway.

By using the measured data from strain QL158 (without carbon-rewiring strategies) and strain QL58 (with carbon-rewiring strategies) during glucose-limited fed-batch fermentation, an FBA analysis revealed that carbon flux was redistributed among glycolysis, PPP, and AAA biosynthesis pathway, when implementing the PHK pathway-based carbon-rewiring strategies (Fig. 6c). In particular, these results showed that our strategies enabled about a twofold increase of the available carbon flux toward E4P and AAA biosynthesis. Finally, in a fed-batch fermentation, a titer of 10.4 g L^−1^ was achieved, an experiment that, when replicated by using a diploid strain with the same genetic modifications, produced a titer of 12.5 g L^−1^ *p*-HCA (Fig. 6a). Although further optimization may well be required, we are confident that the carbon-rewiring strategies presented here provide a promising platform for the production of many other flavonoids and alkaloids derived from phenylalanine and tyrosine.

## Methods

### Construction of strains and reagents

*E. coli* DH5α was used for the construction and propagation of all plasmids. All plasmids and *S. cerevisiae* strains used in this study are listed in Supplementary Data 1 and 3. High-fidelity Phusion DNA polymerase and Gibson assembly kit were purchased from New England Biolabs (Ipswich, MA, USA). PrimeStar DNA polymerase and SapphireAmp^®^ Fast PCR Master Mix were purchased from TaKaRa Bio (Kusatsu, Shiga, Japan). Restriction enzymes, plasmid miniprep, and DNA gel purification kits were purchased from Thermo Fisher Scientific (Waltham, MA, USA). All codon-optimized heterologous genes were provided by GenScript (Nanjing, China) and listed in Supplementary Data 5. All oligonucleotides (Supplementary Data 6) were synthesized at Sigma-Aldrich (St. Louis, MO, USA). All chemicals including analytical standards were purchased from Sigma-Aldrich (St. Louis, MO, USA) unless stated otherwise.

### Strain cultivation

Yeast strains for preparation of competent cells were cultivated in YPD consisting of 20 g L^−1^ peptone (BD Difco^TM^, Thermo Fisher Scientific, Waltham, MA, USA), 10 g L^−1^ yeast extract (Merck Millipore, Burlington, MA, USA), and 20 g L^−1^ glucose (VWR, Radnor, PA, USA). Strains containing *URA3*-based plasmids were selected on synthetic complete medium without uracil (SC-URA), which consisted of 6.7 g L^−1^ yeast nitrogen base (YNB) without amino acids (Formedium^TM^, Norfolk, UK), 0.77 g L^−1^ complete supplement mixture without uracil (CSM-URA, Formedium^TM^, Norfolk, UK), 20 g L^−1^ glucose (VWR, Radnor, PA, USA), and 20 g L^−1^ agar (Merck Millipore, Burlington, MA, USA). The *URA3* marker was removed and selected against on SC with 5-fluoroorotic acid (SC + 5-FOA) plates containing 6.7 g L^−1^ YNB, 0.77 g L^−1^ complete supplement mixture, and 0.8 g L^−1^ 5-FOA.

Shake-flask batch fermentations for the production of *p*-coumaric acid, resveratrol, and aromatic compounds were performed in minimal medium. Three independent single colonies, with the relevant genetic modifications, were inoculated into 14-mL tubes with 1.5 mL of fresh minimal medium (7.5 g L^−1^ (NH_4_)_2_SO_4_, 14.4 g L^−1^ KH_2_PO_4_, 0.5 g L^−1^ MgSO_4_·7H_2_O, and 20 g L^−1^ glucose), 2 mL L^−1^ trace metal (3.0 g L^−1^ FeSO_4_·7H_2_O, 4.5 g L^−1^ ZnSO_4_·7H_2_O, 4.5 g L^−1^ CaCl_2_·2H_2_O, 0.84 g L^−1^ MnCl_2_·2H_2_O, 0.3 g L^−1^ CoCl_2_·6H_2_O, 0.3 g L^−1^ CuSO_4_·5H_2_O, 0.4 g L^−1^ Na_2_MoO_4_·2H_2_O, 1.0 g L^−1^ H_3_BO_3_, 0.1 g L^−1^ KI, and 19.0 g L^−1^ Na_2_EDTA·2H_2_O), and 1 mL L^−1^ vitamin solutions (0.05 g L^−1^ d-biotin, 1.0 g L^−1^ d-pantothenic acid hemicalcium salt, 1.0 g L^−1^ thiamin–HCl, 1.0 g L^−1^ pyridoxin–HCl, 1.0 g L^−1^ nicotinic acid, 0.2 g L^−1^ 4-aminobenzoic acid, and 25.0 g L^−1^ *myo*-inositol)^45^ supplemented with 60 mg L^−1^ uracil if needed. Tubes were incubated at 30 °C with 200-rpm agitation overnight. Precultures were then inoculated into a 125-mL non-baffled flask carrying 20 mL of minimal medium at an initial optical density measured at 600 nm (OD600) of 0.05 and cultivated at 200 rpm, 30 °C for 72 h. When shake-flask fermentations were conducted to mimic fed-batch conditions, six tablets of FeedBeads^46^ (SMFB08001, Kuhner Shaker, Basel, Switzerland), corresponding to 20 g L^−1^ glucose, were used as the sole carbon source and cultivated for 96 h at 30 °C with 200-rpm agitation. To induce the transcription of genes under the control of *GAL* promoters, 1% galactose was supplemented into the medium as the gratuitous inducer. For fermentation of strains harboring *gal80* deletion, no galactose was added.

Bioreactor cultivations for *p*-HCA production were performed in 1.0-L vessels by using the DasGip Parallel Bioreactors System (DasGip, Germany). The initial batch fermentation was started with 0.25 L of minimal medium containing 20 g L^−1^ glucose. By using a DasGip Control 4.0 System, the temperature was controlled at 30 °C, and the initial agitation was set to 800 rpm and increased to maximally 1200 rpm depending on the dissolved oxygen level, which was maintained above 40% via stirrer speed and gas flow rate by using the DasGip control system. The aeration was controlled by using a DasGip MX4/4 module and initially provided at 36 L h^−1^. The pH was maintained at 5.6 by automatic addition of 4 M KOH and 2 M HCl. The exhaust gas was monitored by using a DasGip Offgas Analyzer GA4. Addition of the acid, base, and glucose feeding was conducted with DasGip MP8 multi-pump modules (pump head tubing: 0.5-mm ID, 1.0-mm wall thickness). During the fed-batch phase, the cells were fed with a 200 g L^−1^ glucose solution with a feed rate that exponentially increased (µ set to 0.05 h^−1^) to maintain a constant biomass-specific glucose consumption rate. The initial feeding rate was calculated by using the biomass yield and concentration that were obtained during prior duplicate batch cultivations with these strains. The feeding was initiated after the CO_2_ levels dropped indicating that glucose was depleted. Physiological parameters and fluxes (*q*) were calculated based on samples taken during the glucose-limited feeding phase, with the biomass composition of CH_1.8_O_0.5_N_0.2_ being assumed.

Dry cell weight (DCW) measurements were performed by filtering 5 mL of broth through a preweighed 0.45-µm filter membrane (Sartorius Biolab, Göttingen, Germany). The filter was washed once before and three times after filtering the broth with 5 mL of deionized water. All filters were heated in the microwave oven for 15 min and dried for 2 days in a desiccator before measuring the weight increase. During the fermentation process, crystals of *p*-HCA were found to form and accumulate on the filters for measuring DCW. For the imaging of *p*-HCA crystals, 3 μl of QL58 cell cultures cultivated in the bioreactor for 96 h were dropped onto a microscope slide and viewed with a LEICA DM2000 fluorescence microscope (Leica Microsystems GmbH, Wetzlar, Germany) with a ×100 oil objective and processed with the Lecia Application Suite software.

### Genetic manipulation

*S. cerevisiae* strain CEN.PK113-5D-derivative IMX581 (*MATa ura3-52 can1∆::cas9-natNT2 TRP1 LEU2 HIS3*) was used as the background strain for all genetic manipulations. IMX581 harbors an integrated Cas9 expression cassette under the control of constitutive *TEF1* promoter^47^. For gene overexpression, DNA integration constructs were produced and integrated at selected genomic loci via the *CRISPR/cas9* system. All native promoters, genes, and terminators were PCR amplified by using IMX581 genomic DNA as the template. For optimized heterologous genes, synthetic fragments (obtained from GenScript) or plasmids were used for PCR amplification (Supplementary Data 1). High-fidelity Phusion DNA polymerase was utilized throughout the entire molecular cloning procedure, except PrimeSTAR HS polymerase was employed for in vitro DNA part fusion and module generation. Functional expression modules were generated according to overlapping extension PCR (OE-PCR) procedure^48^. All used integration cassettes are listed in Supplementary Data 2.

All gene overexpression cassettes were integrated into designed chromosomal loci that have been demonstrated to provide stable and high-level expression of heterologous genes^49^. By taking the example of targeted integration of M1 at the *XII-2* locus in strain IMX581, the whole pathway was divided into two fragments: I (*XII-2 us-GPM1p-AtPAL2-FBA1t-CYC1t-AtC4H-TDH3p-tHXT7p*) and II (*TDH3p-tHXT7p-AtATR2-pYX212t-ADH1t-CYB5-PGK1p-XII-2 ds*). Fragment I was assembled by fusing DNA from *XII-2 us*, *GPM1p*, *AtPAL2*, *FBA1t-CYC1t*, *AtC4H*, and *TDH3p-tHXT7p*. In detail, the upstream homologous arm *XII-2 us* (Supplementary Data 4) and *GPM1p* were amplified from IMX581 genomic DNA with primer pairs P92/P93 and P1/P2, respectively; the bidirectional promoters *TDH3p-tHXT7p* and terminators *FBA1t-CYC1t* were amplified from pFab1 by using primer pairs P9/P11 and P3/P6, respectively; genes *AtPAL2* and *AtC4H* were amplified from pCfB1018 by using primer pairs P36/P38 and P40/P42, respectively. Fragment II was assembled by fusing the DNA parts of *TDH3p-tHXT7p*, *AtPAL2*, *pYX212t*, *ADH1t*, *CYB5*, *PGK1p,* and *XII-2 ds*. Genes *AtATR2* and *CYB5* were amplified from pCfB848 by using primer pairs P43/P44 and P45/P46, respectively; promoter *PGK1p* was amplified from pFab1 by using primer pairs P16/P17; terminators *pYX212t* and *ADH1t* were amplified from pFab1 by using primer pairs P12/P13 and P14/P15, respectively; the downstream homologous arm *XII-2 ds* (Supplementary Data 4) was amplified from IMX581 genomic DNA with primer pair P94/P95. To construct the fusion protein of 4-coumarate-CoA ligase 1 (*At4CL1*) and stilbene synthase (*VvSTS*), one copy of a flexible linker GSG was used. Then co-transformation of equimolar amounts of purified fragments I and II (50–100 ng/kb) with gRNA plasmid pQC007 (~300–500 ng) into *S. cerevisiae* was conducted according to the described protocol^47^, and transformants were selected on SC-URA plates. Clones were verified by colony PCR by using SapphireAmp^®^ Fast PCR Master Mix. Subsequently, three clones with correct module integration were cultivated overnight in YPD liquid medium and then streaked onto SC + FOA plates after washing to loop out of gRNA vectors and recycle the *URA3* marker. For gene deletion, 2 µg of a double-stranded DNA fragment consisting of two 60-bp sequences homologous to up‐ and downstream regions of the chromosomal target site was used for the homologous repair of the genome double-strand break introduced by cleavage of the Cas9 nuclease. For the construction of the promoter-substitution strain library, the native promoters of *PFK1* (from −162 to 0 bp), *PFK2* (from −63 to 0 bp), and *PYK1* (from −150 to 0 bp) were replaced by selected promoters in Supplementary Table 1 by using the *CRISPR/cas9* system. For the construction of the diploid strain, *CRISPR/cas9*-based mating-type switching^50^ was employed, and PCR was performed to identify correct transformants by using mating-type specific primer pairs^51^. To render galactose as a gratuitous inducer for the activation of *GAL* promoters, genes *GAL7/10/1* were deleted in the QL36 background. Furthermore, deletion of *GAL80* was introduced into the QL57 background to enable induction of *GAL* promoters without the addition of galactose under FeedBeads-based shake-flask cultivations and in glucose-limited fed-batch bioreactor fermentations. The schematic overview of construction of strains is shown in Supplementary Fig. 1.

To select for specific guide RNAs, all potential gRNAs for a selected gene/genomic locus were compared with all potential off-targets in the entire CEN.PK113-7D genome by using CRISPRdirect tool^52^, a freely available tool provided at http://crispr.dbcls.jp/. All single and double gRNA plasmids were constructed following in vitro Gibson assembly method in which gRNA sequence-containing DNA fragments were recombined with vector backbone^47^. Two triple gRNA plasmids pQC156 and pQC185 were constructed by Gibson assembly in which the donor single and receptor double gRNA vectors were involved. Specifically, gRNA expression cassettes for *PYK1p* and *GAL80* were amplified from pQC117 and pQC003, respectively, by primers P262 and P263. The resultant fragments were gel-purified and ligated with *Eco*RV-digested receptor plasmids pQC120 and pQC184, respectively. Correct recombinant plasmids were then verified by sequencing.

### Promoter-substituted library construction and identification

To identify a suitable starting strain for the construction of the promoter screening library, the growth and betaxanthin accumulation profiles of strains QL44–QL47 and the parental strain IMX581 were evaluated on both solid SC plates and liquid minimal media. Overnight cultures were harvested, and the resultant cell pellets were washed twice with fresh minimal medium. For the spot test, about 1 × 10^5^ cells were used, and the initial OD600 value for liquid was kept at 0.05. Both plates and tubes were photographed at shown time intervals (Supplementary Fig. 11).

For screening of promoter-substitution strains, 30 replacement cassettes (Supplementary Data 2) and a triple gRNA vector pQC156 were co-transformed into strain QL45. Transformants were then plated on four SC-URA plates at a density of ~500 colonies per plate. Approximately 300 colonies that appeared to have a relatively higher color intensity compared with the rest of the population were selected and inoculated into 1 mL of minimal medium and grown for 24 h at 30 °C with 200-rpm agitation. The final OD values and color change of the resulting cultures were then compared with that of the background strain QL45 in the first round of screening. Subsequently, the eight most intensely yellow-pigmented colonies were isolated for further growth analysis and identified for their promoter integration profile. For the evaluation of betaxanthin accumulation by using FeedBeads as the sole carbon source, overnight cultures of selected strains were transferred to 20 mL of fresh minimal medium (OD600 = 0.05, six tablets of FeedBeads SMFB08001) and cultivated at 30 °C with 200-rpm agitation. One-hundred microliter cell cultures were transferred to microplates and measured for OD600 and fluorescence^37^ (excitation: 485 nm, emission: 520 nm, and gain: 200) in a FLUOstar Omega microplate reader (BMG Labtech, Ortenberg, Germany) with an interval of 12 h. The background fluorescence measurements of parental strain IMX581 were subtracted to determine their final fluorescence intensity.

### Metabolite extraction and quantification

*p*-HCA and resveratrol production in whole-cell cultures were quantified by high-performance liquid chromatography (HPLC)^30,44^. Specifically, 0.5 mL of cell culture was mixed with an equal volume of absolute ethanol (100% v/v), vortexed thoroughly, and centrifuged at 13,500 × *g* for 5 min. The supernatants were analyzed on a Dionex Ultimate 3000 HPLC (Thermo Fisher Scientific, Waltham, MA, USA) equipped with a Discovery HS F5 150-mm × 4.6-mm column (particle size 5 µm) (Sigma-Aldrich, St. Louis, MO, USA) connected to a photodiode array detector. The column was kept at 30 °C, and metabolites from 10 µL of supernatants were separated. For *p*-HCA and resveratrol detection, a flow rate set to 1.5 mL min^−1^ was used. Samples were analyzed by using a gradient method with two solvents: 10 mM ammonium formate, pH 3.0 (A) and acetonitrile (B). The program started with 5% of solvent B (0–0.5 min), after which its fraction was increased linearly from 5% to 60% (0.5–9.5 min), then the fraction was increased from 60% to 100% (9.5–10.5 min) and maintained at 100% for 0.5 min (10.5–11 min), and at last the fraction was decreased from 100% to 50% (11–11.5 min) and maintained at 5% for 1.5 min (11.5–13 min). *p*-HCA was detected at 6.3 min (304 nm), resveratrol at 7.6 min (333 nm).

The production of aromatic compounds was quantified by HPLC^14,24^. One millilliter of culture broth was centrifuged at 13,500 × *g* for 5 min, and the supernatant was frozen at −20 °C. An aliquot of 400 µL of the culture supernatant was mixed with an equal volume of absolute ethanol (100% v/v), vortexed thoroughly, and centrifuged at 13,500 × *g* for 5 min. The supernatants were used for analysis of aromatic compounds. Two elution systems were used to detect the aromatic compounds. For *p*-hydroxyphenylacetate, *p*-hydroxyphenylethanol, *p*-hydroxyphenylpyruvate, tryptophol, and chorismate, samples were analyzed by using a gradient method with two solvents: water with 0.1% formic acid (A) and acetonitrile (B) at a flow rate of 1.2 mL min^−1^. The program started with 5% of solvent B (0–0.5 min), after which its fraction was increased linearly from 5% to 60% (0.5–18.5 min), then the fraction was maintained at 60% (18.5–20.5 min), after that the fraction was decreased from 60% to 5% (20.5–21 min), and finally, the fraction was maintained at 5% (21–22 min). *p*-Hydroxyphenylacetate was detected at 7.5 min (276 nm), *p*-hydroxyphenylethanol at 6.5 min (276 nm), *p*-hydroxyphenylpyruvate at 10.2 min (304 nm), tryptophol at 11.5 min (276 nm), and chorismate at 6.1 min (276 nm). For phenylethanol and phenylacetate, samples were analyzed by using a gradient method with two solvents: 20 mM KH_2_PO_4_ (pH 3.0) with 1% acetonitrile (A) and acetonitrile (B) at a flow rate of 1 mL min^−1^. The program increased from 0% to 10% solvent B (0–6.0 min), after which its fraction was increased linearly from 10% to 60% (6.0–25 min), then the fraction was decreased from 60% to 0% (25–26 min), and finally, the fraction was maintained at 0% for 2 min (26–28 min). Phenylethanol was detected at 15.5 min (215 nm) and phenylacetate at 17.3 min (215 nm). Authentic standards were used to generate the calibration curves for quantification of *p*-coumaric acid, resveratrol, and production of the other aforementioned aromatic compounds. The yield of *p*-HCA was calculated by dividing the mass of the product formed by the total mass of carbon (glucose) supplied at the time of culture inoculation.

The concentrations of residual glucose, ethanol, glycerol, organic acids (acetate, pyruvate, and succinate), and shikimate were determined by HPLC analysis^53^. Briefly, a 1.0-mL culture sample was centrifuged and filtered through a 0.45-µm syringe filter and analyzed on an Aminex HPX-87G column (Bio-Rad, Hercules, CA, USA) on an Ultimate 3000 HPLC. The column was eluted with 5 mM H_2_SO_4_ at a flow rate of 0.6 mL min^−1^ at 45 °C for 35 min.

### In silico analyses

In order to perform in silico simulations, we reconstructed an enzyme-constrained model of yeast. The model is an extension of ecYeast7^54^ with addition of the heterologous *p*-HCA formation pathways including both the *TAL* and *PAL* branches. As well, we collected turnover rates for all the added enzymes from the BRENDA website^55^ to impose enzyme usage constraints for these reactions (Supplementary Data 7).

To estimate the maximum theoretical yield of *p*-HCA without concurrent biomass formation or maintenance, both growth rate and the non-growth-associated maintenance were set to zero. Besides, we set the upper bound of the glucose uptake rate to 1000 mmol g DCW^−1^ h^−1^ and maximized the production rate of *p*-HCA. The calculated glucose uptake rate cannot reach the upper bound due to the limited protein content in the enzyme-constrained model^54^. The maximum theoretical yield was calculated by the *p*-HCA production rate over the calculated glucose uptake rate.

To estimate the flux distributions, we constrained the related exchange reaction rates to the measured data (Supplementary Table 3), except growth rate, which was maximized instead. We performed flux balance analysis (FBA) to solve the linear programming problem. The estimated growth rates were then confirmed to be consistent with the measured values for growth rate (Supplementary Data 8). All simulations were performed in MATLAB with the COBRA toolbox^56^. All code used in the study is available in Supplementary Data 9.

### Reporting summary

Further information on research design is available in the Nature Research Reporting Summary linked to this article.

## Supplementary information

Supplementary Information

Peer Review

Reporting Summary

Description of Additional Supplementary Files

Supplementary Dataset 1

Supplementary Dataset 2

Supplementary Dataset 3

Supplementary Dataset 4

Supplementary Dataset 5

Supplementary Dataset 6

Supplementary Dataset 7

Supplementary Dataset 8

Supplementary Dataset 9

## Data Availability

Data supporting the findings of this study are available within the article and its Supplementary Information files. A reporting summary for this article is available as a Supplementary Information file. The datasets generated and analyzed during this study are available from the corresponding author upon request. The source data underlying Figs. 2b–d, 3b–d, 4b, 4c, 5b, 5c, 6a, and 6b, as well as Supplementary Figs. 3–6, 8–10, 12c, 15–18, and 19b are provided as a Source Data file.

## Source Data

Source Data
