# Supplementary material for: Rewiring carbon metabolism in yeast for high level production of aromatic chemicals
Source: Nat Commun. 2019 Oct 31;10:4976. doi: 10.1038/s41467-019-12961-5 (PMC6823513; doi:10.1038/s41467-019-12961-5)
Supplement: Supplementary file 1 — Supplementary Information [file 41467_2019_12961_MOESM1_ESM.pdf]

Rewiring carbon metabolism in yeast for high level production of aromatic chemicals

Liu *et al.*

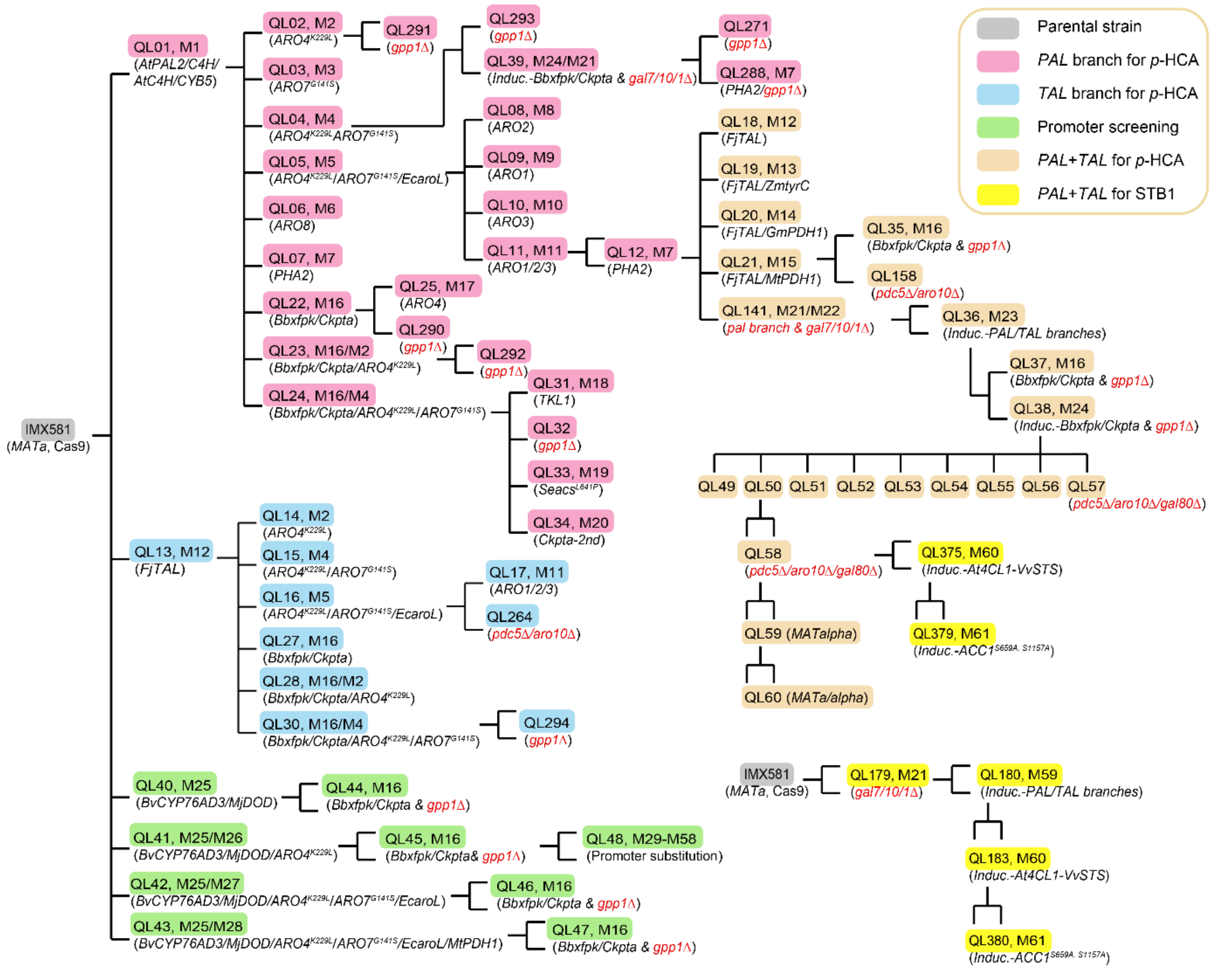

Supplementary Fig. 1. Flowchart of yeast strain construction in this study.

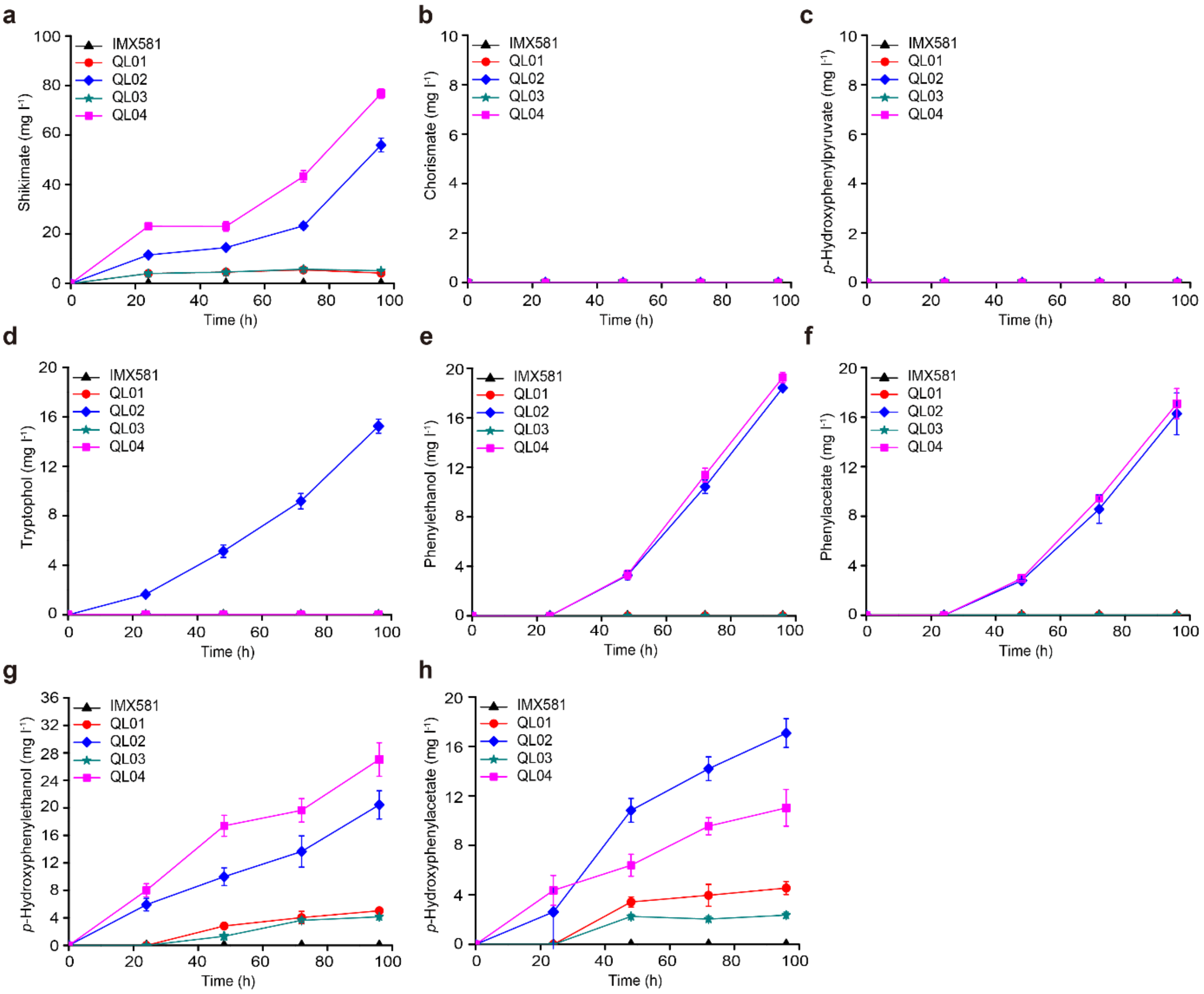

Supplementary Fig. 2. Extracellular concentrations of metabolite intermediates produced by engineered *p*-HCA producing strains. Production of (**a**) Shikimate, (**b**) Chorismate, (**c**) *p*-Hydroxyphenylpyruvate, (**d**) Tryptophol, (**e**) Phenylethanol, (**f**) Phenylacetate, (**g**) *p*-Hydroxyphenylethanol and (**h**) *p*-Hydroxyphenylacetate by strains QL01, QL02, QL03, QL04 and the wild type strain IMX581 were shown following time points. For shake flask cultivation, cells were grown in defined minimal medium with 6 tablets of FeedBeads as the sole carbon source, and cultures were sampled every 24 h for metabolites detection. All data represent the mean of n = 3 biologically independent samples and error bars show standard deviation.

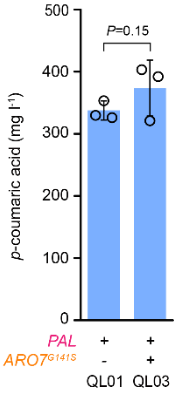

Supplementary Fig. 3. Effect on *p*-HCA production of overexpressing the L-tyrosine-feedback insensitive mutant *ARO7*^{G141S} via the *PAL* branch. Cells were grown in defined minimal medium with 6 tablets of FeedBeads as the sole carbon source, and cultures were sampled after 96 h of growth for *p*-HCA detection. Statistical analysis was performed using one-tailed Student's t test (one-tailed; two-sample unequal variance; *$p < 0.05$, **$p < 0.01$, ***$p < 0.001$). All data represent the mean of n = 3 biologically independent samples and error bars show standard deviation. Source data are provided as a Source Data file.

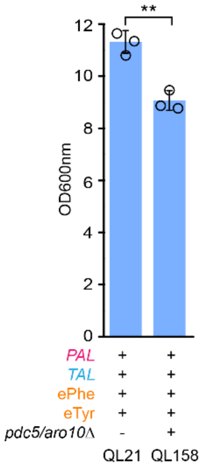

Supplementary Fig. 4. Removal of the AAA degradation pathway reduced cell growth. For shake flask cultivation, cells were grown in defined minimal medium with 6 tablets of FeedBeads as the sole carbon source, and cultures were sampled after 96 h of growth for OD600 measurement. Statistical analysis was performed using Student's t test (one-tailed; two-sample unequal variance; *$p < 0.05$, **$p < 0.01$, ***$p < 0.001$). All data represent the mean of n = 3 biologically independent samples and error bars show standard deviation. Source data are provided as a Source Data file.

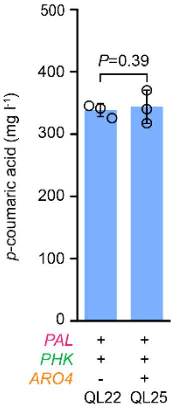

Supplementary Fig. 5. Effects on *p*-HCA production of overexpressing wild-type *ARO4* via the *PAL* branch. Cells were grown in defined minimal medium with 6 tablets of FeedBeads as the sole carbon source, and cultures were sampled after 96 h of growth for *p*-HCA detection. Statistical analysis was performed using one-tailed Student's t test (one-tailed; two-sample unequal variance; $*p < 0.05$, $**p < 0.01$, $***p < 0.001$). All data represent the mean of n = 3 biologically independent samples and error bars show standard deviation. Source data are provided as a Source Data file.

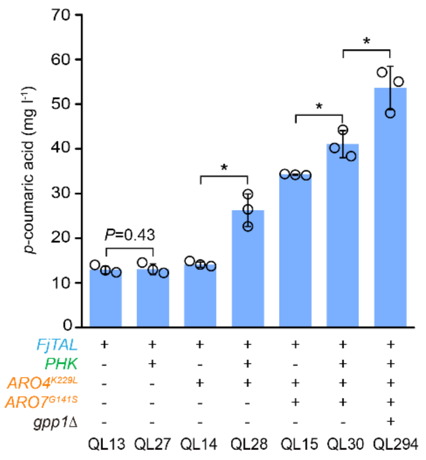

Supplementary Fig. 6. Introduction of the PHK pathway enables a substantial increase in *p*-HCA titers via the *TAL* branch. Cells were grown in defined minimal medium with 6 tablets of FeedBeads as the sole carbon source, and cultures were sampled after 96 h of growth for *p*-HCA detection. Statistical analysis was performed using one-tailed Student's t test (one-tailed; two-sample unequal variance; $*p < 0.05$, $**p < 0.01$, $***p < 0.001$). All data represent the mean of n = 3 biologically independent samples and error bars show standard deviation. Source data are provided as a Source Data file.

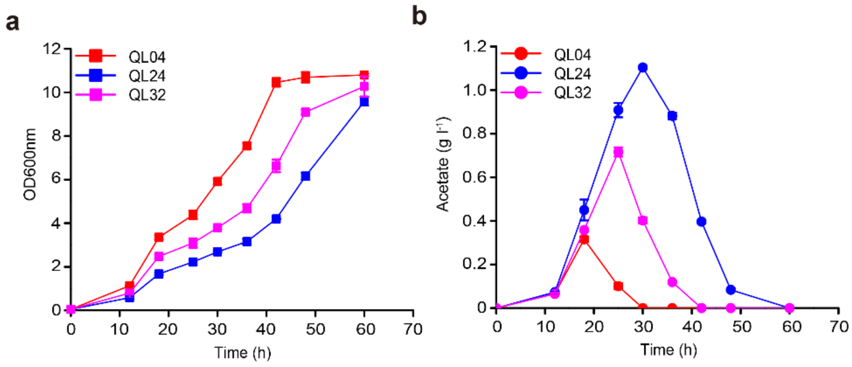

Supplementary Fig. 7. Deletion of *GPP1* gene reduces the accumulation of acetate with improved cell growth in PHK pathway-expressing strains. Optical density measurements (**a**) and time course of acetate accumulation (**b**) during the whole growth phase are shown for three engineered strains, including the PHK-negative strain QL04 (red symbols), the PHK-expressing strain QL24 (blue symbols) and strain QL32 (pink symbols) carrying both the integrated PHK pathway and the deletion of *GPP1*. Strains were grown in shake flasks with defined minimal medium containing 2% glucose. All data represent the mean of n = 3 biologically independent samples and error bars show standard deviation.

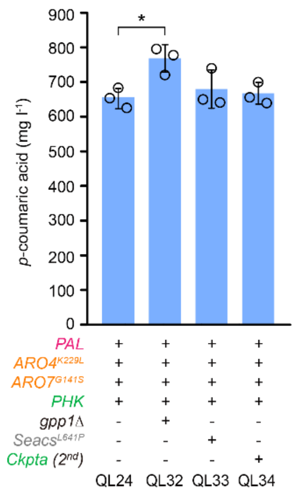

Supplementary Fig. 8. Optimization of the PHK pathway for *p*-HCA production via the *PAL* branch. Different strategies, including (i) deleting the native glycerol-1-phosphatase encoding gene *GPP1*, (ii) overexpressing acetyl-CoA synthetase mutant (SeAcs^{L641P}) from bacterium *Salmonella enterica*, or (iii) introducing an additional copy of phosphotransacetylase (CkPta), were compared. Cells were grown in defined minimal medium with 6 tablets of FeedBeads as the sole carbon source, and cultures were sampled after 96 h of growth for *p*-HCA detection. Statistical analysis was performed using Student's t test (one-tailed; two-sample unequal variance; **p* < 0.05, ***p* < 0.01, ****p* < 0.001). All data represent the mean of n = 3 biologically independent samples and error bars show standard deviation. Source data are provided as a Source Data file.

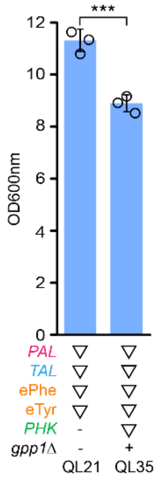

Supplementary Fig. 9. Increased *p*-HCA production via phosphoketolase pathway expression leads to decreased cell biomass. Open triangles indicate using constitutive strong promoters to control gene expression. Cells were grown in defined minimal medium with 6 tablets of FeedBeads as the sole carbon source, and cultures were sampled after 96 h of growth for optical density evaluation. Statistical analysis was performed using one-tailed Student's t test (one-tailed; two-sample unequal variance; *$p < 0.05$, **$p < 0.01$, ***$p < 0.001$). All data represent the mean of n = 3 biologically independent samples and error bars show standard deviation. Source data are provided as a Source Data file.

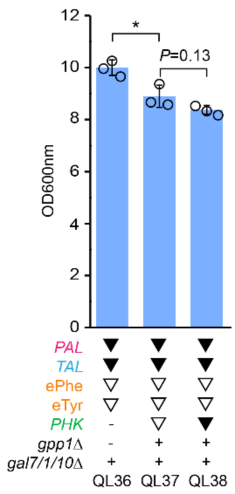

Supplementary Fig. 10. Dynamic control of biosynthetic genes retains cell growth capacity with increased *p*-HCA production. Open triangles indicate using constitutive strong promoters to control gene expression, while filled triangles indicate using galactose-induced promoters. Cells were grown in defined minimal medium with 6 tablets of FeedBeads as the sole carbon source and 1% galactose as inducer, and cultures were sampled after 96 h of growth for optical density evaluation. Statistical analysis was performed using one-tailed Student's t test (one-tailed; two-sample unequal variance; $*p < 0.05$, $**p < 0.01$, $***p < 0.001$). All data represent the mean of n = 3 biologically independent samples and error bars show standard deviation. Source data are provided as a Source Data file.

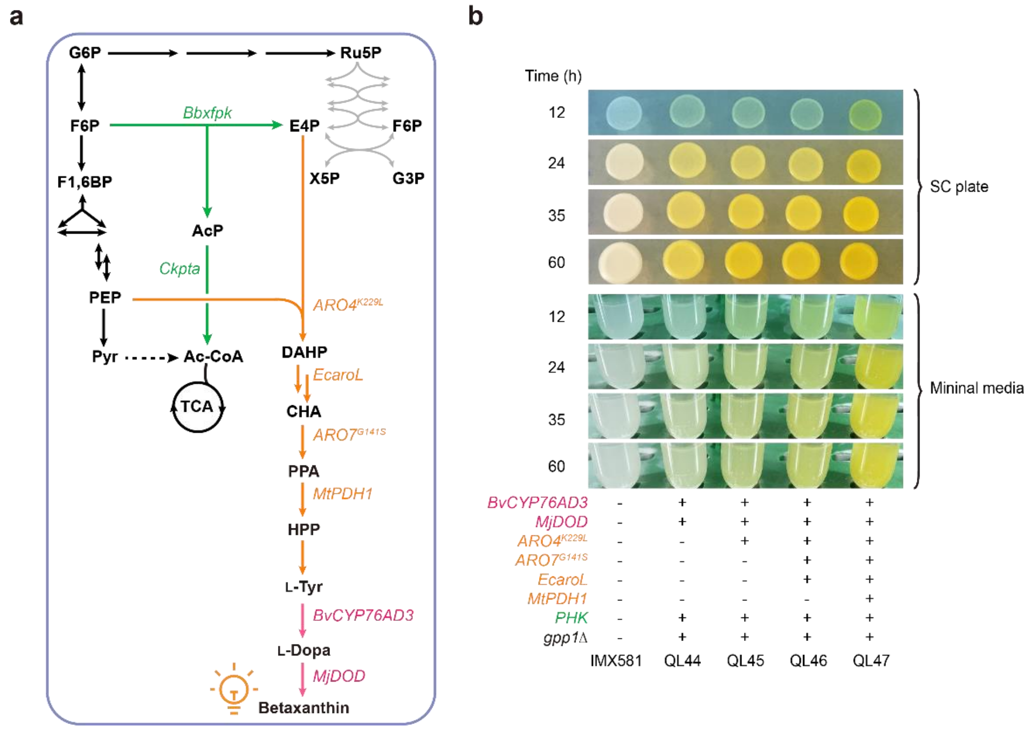

Supplementary Fig. 11. Optimization of the pigment betaxanthin-mediated screening platform. (**a**) Schematic overview of the metabolic pathway for L-tyrosine-derived betaxanthin formation. Two heterologous enzymes, L-tyrosine hydroxylase, encoded by *BvCYP76AD3* variant from *Beta vulgaris,* and L-DOPA dioxygenase, encoded by *MjDOD* from the plant *Mirabilis jalapa*, were expressed to convert invisible L-tyrosine to yellow betaxanthin. Introduction of the PHK pathway (containing BbXfpk and CkPta) combined with feedback insensitive DAHP synthase ($ARO4^{K229L}$), chorismate mutase ($ARO7^{G141S}$), *Escherichia coli* shikimate kinase (*EcaroL*), and prephenate dehydrogenase *MtPDH1* from *Medicago truncatula,* were used as different background strains for the initial screening test. See Fig. 1 and Fig. 2 legends regarding abbreviations of metabolites and more gene information. (**b**) Time courses of color change by engineered betaxanthin producing strains. Approximately 1 x 10^5 cells of indicated strains were spotted on SC plates and incubated at 30 °C. For observing betaxanthin formation under liquid culture conditions, cells were cultured with an initial OD600 of 0.05 in in defined minimal medium with 20 g l^{-1} glucose at 30°C, 200 rpm. Plates and tubes were photographed at the highlighted time intervals.

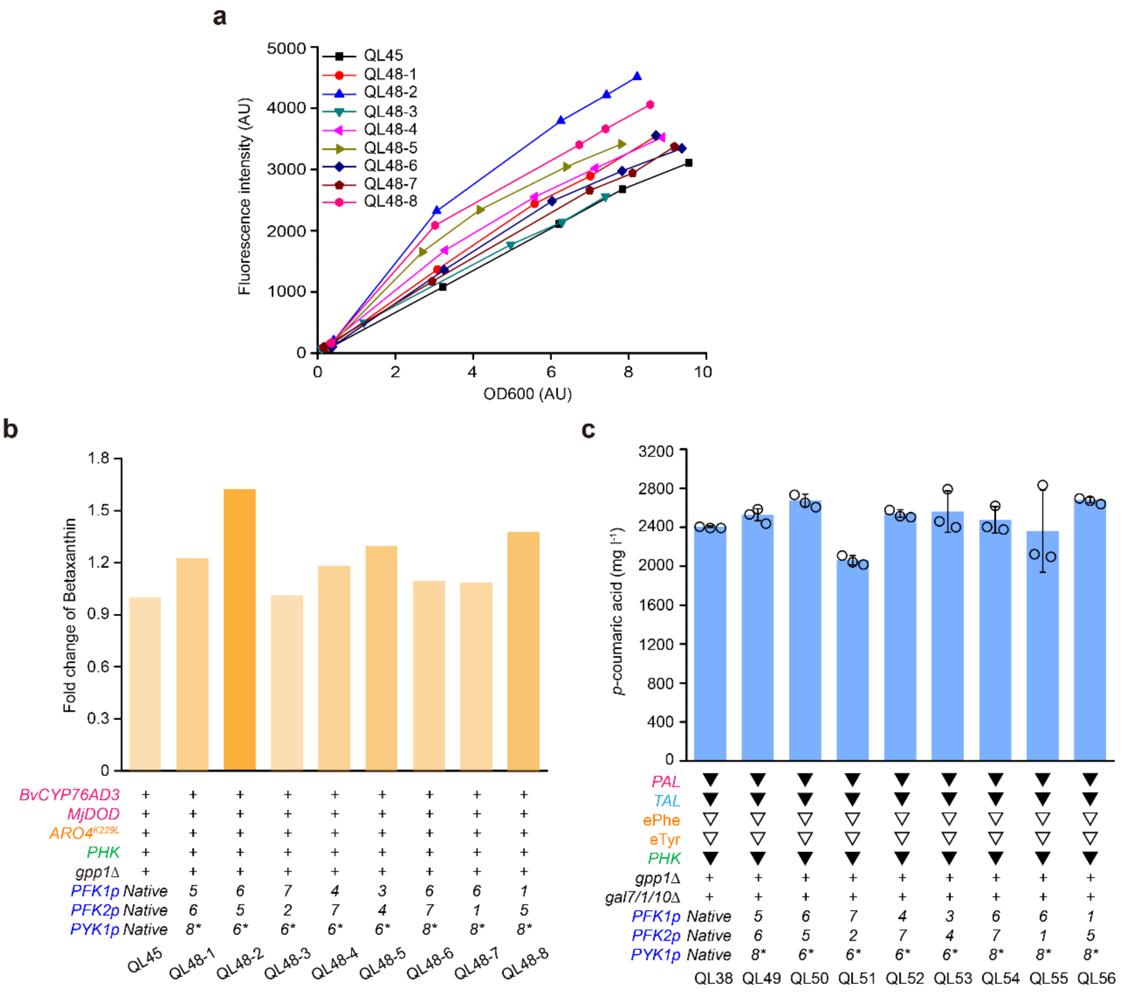

Supplementary Fig. 12. Evaluation of the promoter replaced strains based on betaxanthin screening. (**a**) Cell growth and betaxanthin accumulation in selected promoter-replaced strains. Cells were grown in defined minimal medium with 6 tablets of FeedBeads as the sole carbon source. All data represent the mean of biological triplicates. (**b**) Relative accumulation level of betaxanthin in selected promoter-replaced strains compared with strain QL45 as the reference. The values denote the normalized fold change of betaxanthin accumulation per cell mass increase. The color change in bars reflects increase in betaxanthin formation. Exact promoters in selected promoter-replaced strains are indicated as corresponding numbers listed in Supplementary Table 2. (**c**) Reconstitution of the promoter replacement in engineered *p*-HCA producer (QL38) background. Open triangles indicate using constitutive strong promoters to control gene expression, while filled triangles indicate using galactose-induced promoters. Cells were grown in defined minimal medium with 6 tablets of FeedBeads as the sole carbon source and 1% galactose as inducer. All data represent the

mean of n = 3 biologically independent samples and error bars show standard deviation. The source data underlying Supplementary Figure 12c are provided as a Source Data file.

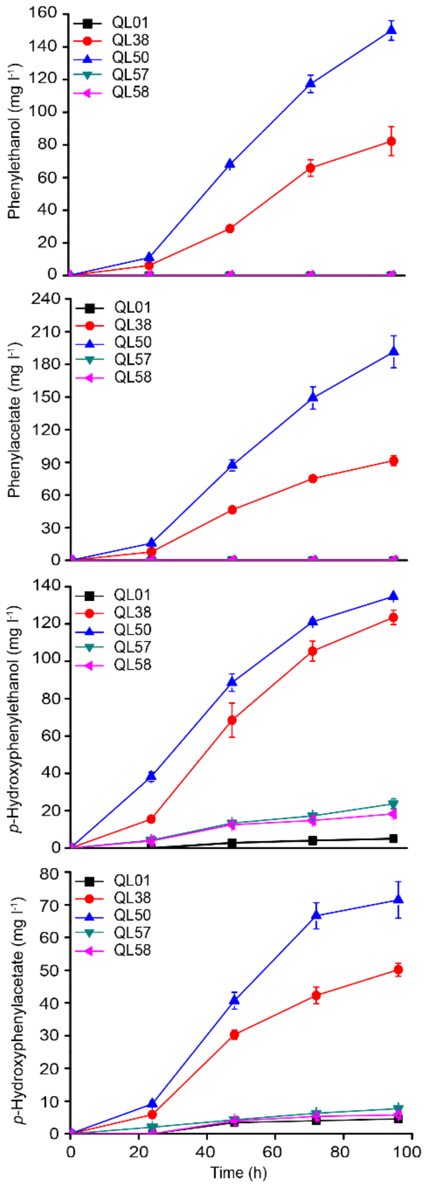

Supplementary Fig. 13. Deletion of *PDC5* and *ARO10* genes reduced the formation of extracellular fusel alcohols and corresponding organic acids. Production profiles of phenylethanol, phenylacetate, p-hydroxyphenylethanol and p-hydroxyphenylacetate by strains QL01, QL38, QL50, QL57 and QL58 were shown. For shake flask cultivation, cells were grown in defined minimal medium with 6 tablets of FeedBeads as the sole carbon source and 1% galactose as inducer when required, and cultures were sampled every 24 h for metabolites detection. All data represent the mean of n = 3 biologically independent samples and error bars show standard deviation.

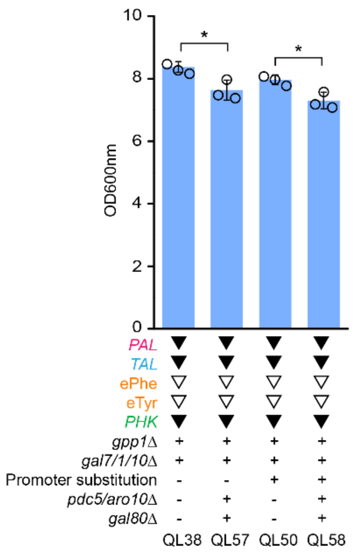

Supplementary Fig. 14. Growth profiles of high-level p-HCA production strains with and without removing the AAA degradation pathway. Open triangles indicate the use of constitutive strong promoters to control gene expression, while filled triangles indicate the use of galactose-inducible promoters. For shake flask cultivation, cells were grown in defined minimal medium with 6 tablets of FeedBeads as the sole carbon source and 1% galactose as the inducer when required. For strains containing a GAL80 deletion, no galactose was supplemented. Cultures were sampled after 96 h of growth for OD600 measurement. Statistical analysis was performed using Student's t test (one-tailed; two-sample unequal variance; $*p < 0.05$, $**p < 0.01$, $***p < 0.001$). All data represent the mean of n = 3 biologically independent samples and error bars show standard deviation.

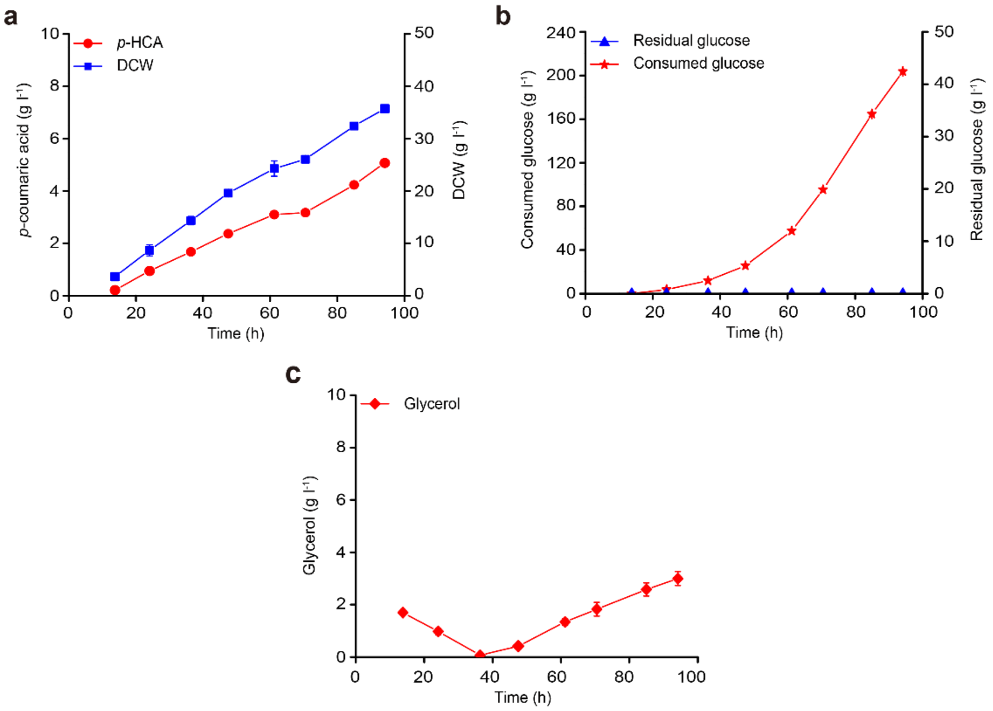

Supplementary Fig. 15. Fed-batch fermentation of strain QL158 under glucose limited conditions. (**a**) Time courses of *p*-HCA titers (red symbols) and cell mass (blue symbols) are shown. (**b**) Glucose consumption profile (red symbols) and time course of residual glucose (blue symbols) during fed-batch fermentation. (**c**) Accumulation of by-product glycerol is shown. All data represent the mean of n = 2 biologically independent samples and error bars show standard deviation. Source data are provided as a Source Data file.

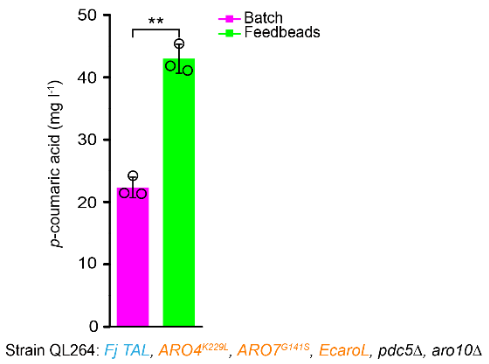

Supplementary Fig. 16. Reconstructed *p*-HCA overproducer strain. *p*-HCA titers obtained with engineered strain derived from the *TAL* branch in shake flasks with 6 tablets of FeedBeads (green) and batch 20 g l^{-1} glucose (purple) as the sole carbon source, respectively, after 96 h cultivation at 30° C, 200 rpm. Statistical analysis was performed using Student's t test (one-tailed; two-sample unequal variance; **p* < 0.05, ***p* < 0.01, ****p* < 0.001). All data represent the mean of n = 3 biologically independent samples and error bars show standard deviation. Source data are provided as a Source Data file.

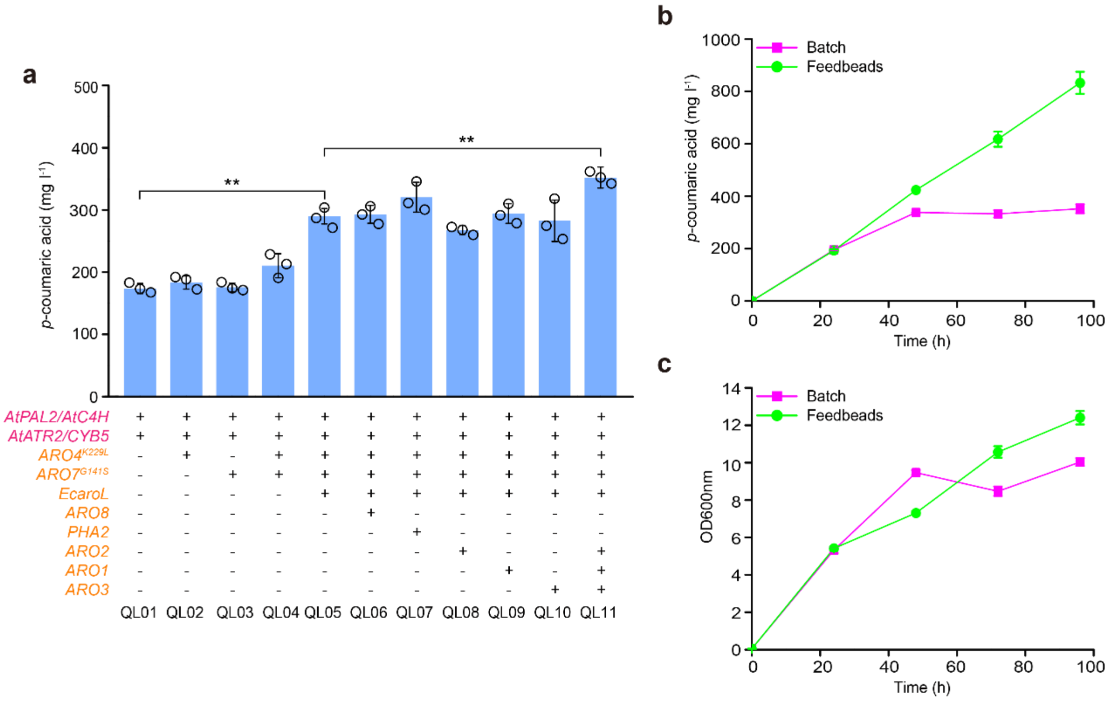

Supplementary Fig. 17. Availability of carbon source determines cell capacity for *p*-HCA overproduction. (**a**) *p*-HCA titers obtained with engineered strains derived from the *PAL* branch in shake flasks with 20 g l^{-1} glucose as carbon source after 96 h cultivation at 30 °C, 200 rpm. Time courses of the *p*-HCA production (**b**) and growth profiles (**c**) of strain QL11 using 6 tablets of FeedBeads (green symbols) and batch 20 g l^{-1} glucose (purple symbols) as the sole carbon source, respectively. Statistical analysis was performed using Student's t test (one-tailed; two-sample unequal variance; **p* < 0.05, ***p* < 0.01, ****p* < 0.001). All data represent the mean of n = 3 biologically independent samples and error bars show standard deviation. Source data are provided as a Source Data file.

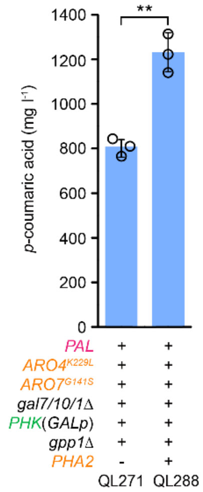

Supplementary Fig. 18. Effect of *PHA2* overexpression on *p*-HCA production. Cells were grown in defined minimal medium with 6 tablets of FeedBeads as the sole carbon source and 1% galactose as inducer, and cultures were sampled after 96 h of growth for *p*-HCA detection. Statistical analysis was performed using Student's t test (one-tailed; two-sample unequal variance; *$p < 0.05$, **$p < 0.01$, ***$p < 0.001$). All data represent the mean of n = 3 biologically independent samples and error bars show standard deviation. Source data are provided as a Source Data file.

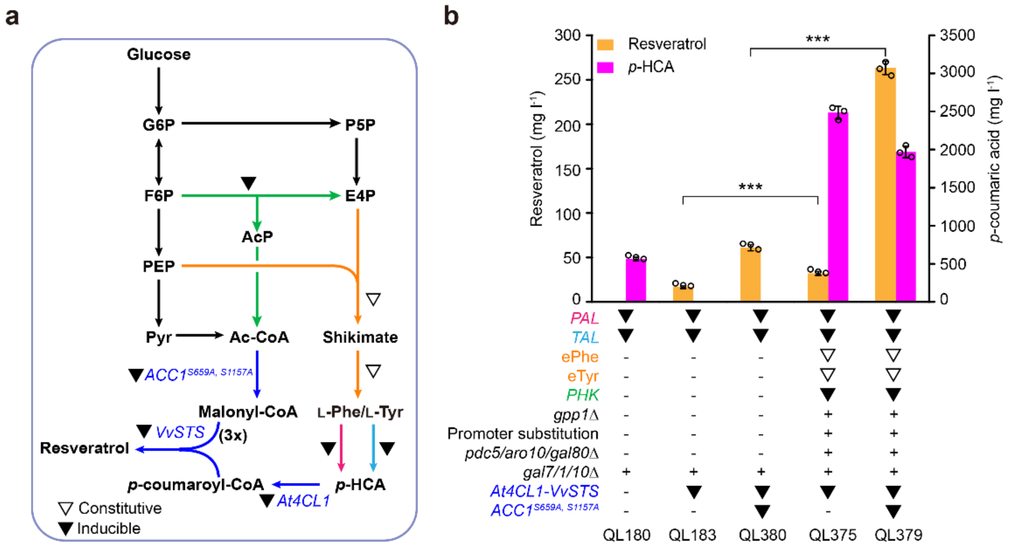

Supplementary Fig. 19. Enabling higher resveratrol production by engineered *p*-HCA biosynthesis. (**a**) Schematic illustration of resveratrol biosynthetic pathway in the context of enhanced supply of precursor *p*-HCA. The resveratrol biosynthetic pathway consists of *Arabidopsis thaliana* 4-coumarate-CoA ligase 1 (*At4CL1*) and *Vitis vinifera* stilbene synthase (*VvSTS*); the deregulated mutant *ACC1*$^{S659A,\ S1157A}$ was overexpressed to increase the supply of another precursor malonyl-CoA. Open triangles indicate use of constitutive strong promoters to control gene expression, while filled triangles indicate use of galactose-induced promoters. (**b**) Resveratrol and *p*-HCA titers obtained with engineered strains. Cells were grown in defined minimal medium with 6 tablets of FeedBeads as the sole carbon source and 1% galactose as inducer when required. For strains with deletion of GAL80, no galactose was supplemented. Cultures were sampled after 96 h of growth for resveratrol and *p*-HCA detection. Statistical analysis was performed using Student's t test (one-tailed; two-sample unequal variance; **p* < 0.05, ***p* < 0.01, ****p* < 0.001). All data represent the mean of n = 3 biologically independent samples and error bars show standard deviation. The source data underlying Supplementary Figure 19b are provided as a Source Data file.

Supplementary Table 1. Selected promoters used for fine-tuning the activities of key glycolytic enzymes in *Saccharomyces cerevisiae*.

No.	Alias	Activity on Glucose[a]	No.	Alias	Activity on Glucose[a]
For *PFK1p*/*PFK2p* substitution			For *PYK1p* substitution		
Native	*PFK1p*/*PFK2p*	100%/83%	*Native*	*PYK1p*	100%
1	*ILV3p*	69%	*1**	*RPS11Bp*	42%
2	*ADH3p*	58%	*2**	*RPL23Ap*	30%
3	*VMA6p*	47%	*3**	*YDJ1p*	33%
4	*BAT1p*	36%	*4**	*PRE2p*	19%
5	*CDC24p*	25%	*5**	*RPA12p*	15%
6	*SET3p*	12%	*6**	*ALD5p*	21%
7	*IDP1p*	6%	*7**	*IPP1p*	12%
8	*RAD1p*	5%	*8**	*LYS20p*	82%
9	*PDR3p*	4%	*9**	*HXT7p*	62%
10	*PDS1p*	3%	*10**	*ACS2p*	37%

[a] The transcriptional activities of selected promoters are obtained from previous report[1].

Supplementary Table 2. Promoter substitution profiles of identified yeast strains.

Betaxanthin strains (QL48-#)	*PFK1* upstream	*PFK2* upstream	*PYK1* upstream	Corresponding *p*-HCA strains
1	*CDC24p*	*SET3p*	*LYS20p*	QL49
2	*SET3p*	*CDC24p*	*ALD5p*	QL50
3	*IDP1p*	*ADH3p*	*ALD5p*	QL51
4	*BAT1p*	*IDP1p*	*ALD5p*	QL52
5	*VMA6p*	*BAT1p*	*ALD5p*	QL53
6	*SET3p*	*IDP1p*	*LYS20p*	QL54
7	*SET3p*	*ILV3p*	*LYS20p*	QL55
8	*ILV3p*	*CDC24p*	*LYS20p*	QL56

Supplementary Table 3. Physiological parameters of engineered *p*-HCA producing strains.[a]

Parameter	QL158	QL58
μ(max) (h^{-1})	0.038 ± 0.0004	0.029 ± 0.001
Y(x/s) (g g^{-1})	0.326 ± 0.014	0.323 ± 0.009
q(Glucose) (mmol $gDCW^{-1}$ h^{-1})	-0.562 ± 0.025	-0.479 ± 0.004
q(Glycerol) (mmol $gDCW^{-1}$ h^{-1})	0.061 ± 0.008	N.D.
q(*p*-coumaric acid) (mmol $gDCW^{-1}$ h^{-1})	0.036 ± 0.0003	0.0672 ± 0.005
q(Biomass) (mmol $gDCW^{-1}$ h^{-1})	1.559 ± 0.014	1.175 ± 0.040
q(CO_2) (mmol $gDCW^{-1}$ h^{-1})	1.469 ± 0.019	1.140 ± 0.071
q(O_2) (mmol $gDCW^{-1}$ h^{-1})	-1.494 ± 0.012	-1.141 ± 0.055
Carbon balance	105 ± 5%	102 ± 3%

[a] Data represent the mean ± SD of n = 2 biologically independent samples.

N.D. not detected.

Supplementary References

1. Keren, L. *et al.* Promoters maintain their relative activity levels under different growth conditions. *Mol. Syst. Biol.* **9**, 701 (2013).
