# Supplementary material for: Rewiring carbon metabolism in yeast for high level production of aromatic chemicals
Source: Nat Commun. 2019 Oct 31;10:4976. doi: 10.1038/s41467-019-12961-5 (PMC6823513; doi:10.1038/s41467-019-12961-5)
Supplement: Supplementary file 4 — Description of Additional Supplementary Files [file 41467_2019_12961_MOESM4_ESM.docx]

**Description of Additional Supplementary Files**

File Name: Supplementary Data 1
Description: Plasmids used in this study.

File Name: Supplementary Data 2
Description: Overview of DNA constructs used in this study.

File Name: Supplementary Data 3
Description: S. cerevisiae strains used in this study.

File Name: Supplementary Data 4
Description: Homology sequences for integration at selected chromosomal loci.

File Name: Supplementary Data 5
Description: Codon optimized genes used in this study.

File Name: Supplementary Data 6
Description: Primers used in this study.

File Name: Supplementary Data 7

Description: Turnover rates collected from the BRENDA website for all heterologous *p*-HCA production pathway enzymes used in this study.

File Name: Supplementary Data 8

Description: Estimated flux distribution for strain QL158 and QL58 based on FBA analysis.

File Name: Supplementary Data 9

Description: Codes used in this study for performing *in silico* analysis.
