# Supplementary material for: Rewiring carbon metabolism in yeast for high level production of aromatic chemicals
Source: Nat Commun. 2019 Oct 31;10:4976. doi: 10.1038/s41467-019-12961-5 (PMC6823513; doi:10.1038/s41467-019-12961-5)
Supplement: Supplementary file 2 — Peer Review [file 41467_2019_12961_MOESM2_ESM.pdf]

Reviewers' comments:

Reviewer #1 (Remarks to the Author):

In this manuscript Liu and co-workers describe the reeingineering of the S. cerevisiae metabolism for the production of p-coumaric acid.

Considering the materials and methods section, the experiments are be reproducible and where appropriate the data has been statistically analyzed. The manuscript presents heaps of data. However, when looking for new and innovative strategies, sth. I would expect from a nature paper, there is not much to find.

Debottlenecking of the AAA synthesis (PPP, glycolysis, shikimate pathway) and precursor balancing - this has already been done for yeast and other species. In addition, p-coumaric acid has been a target compound several metabolic engineering campaigns - also in yeast(s) and other microorganisms, and several studies presenting similar studies are already available. In fact, a metabolic engineering study focusing on the same compound has been already published by the same institute in 2015 (10.1016/j.ymben.2015.08.003). In addition, more recently, an innovative p-coumaric acid biosensor application for yeast has been published – again by the same institute (10.1021/acssynbio.7b00009).

Another concern is the suitability of this strain as platform strain for the microbial production of flavonoids and other Tyr- and Phe-derived compounds claimed in the very last sentence. This strain has been heavily engineered to provide Tyr and Phe by rerouting the carbon flux to E4P and PEP. However, flavonoids require only 1 mol p-coumaric acid per mol product, but 3 mol malonyl-CoA per mol product. When looking into literature it appears as if colleagues working on flavonoids indeed identified malonyl-CoA as limiting for the synthesis of these valuable compounds, not the p-coumaric acid supply.

Taken together, I think that this manuscript presents some interesting aspects, but is more interesting for readers active in the same field and thus more suitable for a specialized journal.

Reviewer #2 (Remarks to the Author):

This article described the assembly of many metabolic engineering strategies into one yeast strain that brought a production of coumaric acid at a titer of ~3.0 g/L in shake-flask and 12.5 g/L in fed-batch fermentation, which represented the highest level that has ever been achieved in producing the aromatic amino acid pathway derivatives. The manuscript was nicely prepared, including novel interesting interventions such as introducing phosphoketolase to improve E4P availability. Considering that the manuscript included many intermediate strains (summarized in the supplementary information), some of the comparisons need clearer explanations.

1. There was confusion between the strain Q12 in Figure 2 and the strain QL01 in Figure 3. My understanding is that the "the PAL strain labeled in pink" in Figure 3b included all the beneficial manipulations shown in the Figure 2b and 2d, resulting in a titer of ~1.1 g/L. However, QL01 showed production of only 330 mg/L in Figure 3b.

Similarly, QL24 in Figure 3b seemed to overexpress the feedback-insensitive mutant Aro4* and Aro7*. Did the "the PAL strain labeled in pink" already have these two manipulations as shown in QL12 of Figure 2b? Or there were additional copies for Aro4* and Aro7* being inserted into QL24?

2. What is the relation between QL290 in Figure 3d (350 mg/L) and QL35 in Figure 4b (~1800 mg/L)? My understanding was that they only differed in the addition of the TAL branch based on the label.

However, this enhancement seemed to be dramatic! This was in great contrast when examining QL12, QL18, and QL21 in Figure 2d. The three strains also differed only in the additional genes involved in the Tyr branch, but the increase was only from ~1.1 g/L to 1.6 g/L.

It looked like all the strains in Figure 3 had relatively low titers. Maybe "the PAL strain labeled in pink" (Figure 3) did not include all the manipulations shown in Figure 2?

3. Page 8, the second paragraph: it was mentioned, "…which demonstrated more than 40% enhancements upon the same introduction of GPP1 deletion and phosphoketolase pathway (strain QL293 and QL32". Looking at Figure 3d, I found that both strains carried the gpp1 deletion. So the 40% enhancement did not come from gpp1 deletion.

4. Page 9, the bottom two sentences: the authors used QL45, which only had the phophoketolase pathway and Aro4*. What was the starting titer of QL45?

The authors used CRISPR technology to introduce the combinatorial promoters for the three target genes and found eight improved variants. How many colonies did they screen? The method section only mentioned 500 colonies per plate.

5. Page 12, Line #4 from the top: it was mentioned, "overexpression of PHA2 alone being able to increase p-HCA production by 50%". Although this 50% enhancement was demonstrated in Figure S13, it was not consistent with the increase observed from QL05 to QL07 (shown in Figure 2b).

6. In Figure S10b, QL48-2 has the promoters designated as "6-5-6". Was this an error since three different promoters "SET3p, CDC24p, and ALD5p" were included in this mutant (Page 10)?

Did SET3p and ILV3p carry similar strengths? How about ALD5p compared to LYS20p?

7. Page 31, Figure 5c: gal80 deletion was not described in the text.

Zengyi Shao agreed to sign to enhance the transparency of the peer review process according to the policy of the journal.

Reviewer #3 (Remarks to the Author):

The manuscript by Liu et al. entitled Rewiring carbon metabolism in yeast for high level production of aromatic chemicals describes metabolic engineering strategies to improve the formation of coumaric acid in Saccharomyces cerevisiae. In addition to approaches that have already published already the study investigate diversion of glycolytic flux through conversion of fructose-6P into E4P and acetyl-P using a phosphoketolase (a subsequent phosphotransacetylase to form acetyl CoA). The combination of old and new genetic modifications led to the construction of strains able to produce 3 g L-1 of coumaric acid, which represent a 50% increase as compare to the best previously published study.

Also impressive, the manuscript is oddly organised, by mixing already demonstrated strategies and new ones, instead of first establishing a reference including all the known beneficial genetic modifications and then evaluate the impact of the new approaches. The makes the manuscript difficult to follow and improvements are reported incrementally from one strain to the next. For instance I do not understand why deletion of PDC5 and ARO10 are only performed in final strains since these modifications were known to impact by-product formation. Additionally the impact of the double deletion is only quantified by increase of coumaric acid concentration. Concentrations of phenylethyl alcohol and its hydroxylated derivative should be presented to the readers.

Overall the information provided is limited to extracellular concentration of coumaric acid. However most of the speculation in the title, in the motivation of the genetic modifications and in the discussion relate to changes in fluxes. But no data are provided to make such claims. The main remaining question then is what impact on glycolytic and pentose phosphate fluxes all these modifications have? This information would bring the manuscript beyond any other publication showing just incremental improvement.
Providing fluxes redistribution would elaborate on statement made in the introduction: On page 4 line 4 "the authors write "Metabolic flux analysis in yeast has shown that the available carbon flux towards E4P is at least one order of magnitude lower than the flux to PEP, even in a strain that is optimized for initial 5 AAA pathway productivity". The manuscript should then conclude on the effect of the genetic rewiring of the central carbon metabolism on redistribution of the carbon fluxes.

There are several points on which I do not fully agree with the authors:
The authors should comment on the industrial application of strains that express genes under galactose induction. Is this realistic, if not what could then be an alternative. Further the cultivation conditions used for strains requiring galactose induction should be introduced in the results section at least specified that these cultures are not typical batch cultivation.
In a similar manner clearly mentioned that strain QL60 (page 10 last sentence) is grown in fedbatch with 200 g L-1 of glucose as mentioned in the materials and methods section. How do you perform the galactose induction ? This is unclear.

In the first paragraph of the results the authors evaluate the effect of the alleviation of the feedback allosteric loop on DAHP synthase and chorismate mutase. Based on level of coumaric acid only it is concluded that what has been considered in several studies as critical modifications, did not have a significant impact. To be able to conclude this the authors do have to produce ta on pathway intermediates as shikimate, chorismate, phenylethanol and hydroxyl phenylethanol, tryptophol and related acids. Based on these information only an accurate conclusion on the impact of these modifications could be drawn.

On page 5 "Clearly, the PAL branch was far more efficient compared with the TAL branch, despite intracellular tyrosine concentrations usually reported as being one order of magnitude higher than that of phenylalanine27", This should be actually thoroughly discussed, several other reports do not support this statement. More accurate quantification of intracellular amino acid concentrations that have not been derived from microtiter plate cultures are publically available.

The reference 1 and 3 do not report that "flavonoids and alkaloids, are widely used as food and feed additives" could you provide suitable reference for it?

Response to Reviewer Comments

We would like to thank the reviewers for their constructive and valuable comments for improving our manuscript. The comments were very helpful in helping us revise the manuscript. Point-by-point of reviewer's comments have been addressed as below.

Reviewer 1

In this manuscript Liu and co-workers describe the reeingineering of the *S. cerevisiae* metabolism for the production of p-coumaric acid.

Considering the materials and methods section, the experiments are be reproducible and where appropriate the data has been statistically analyzed. The manuscript presents heaps of data. However, when looking for new and innovative strategies, sth. I would expect from a nature paper, there is not much to find.

Debottlenecking of the AAA synthesis (PPP, glycolysis, shikimate pathway) and precursor balancing - this has already been done for yeast and other species. In addition, p-coumaric acid has been a target compound several metabolic engineering campaigns - also in yeast(s) and other microorganisms, and several studies presenting similar studies are already available. In fact, a metabolic engineering study focusing on the same compound has been already published by the same institute in 2015 (10.1016/j.ymben.2015.08.003). In addition, more recently, an innovative p-coumaric acid biosensor application for yeast has been published – again by the same institute (10.1021/acssynbio.7b00009).

We thank the reviewer for the constructive comments. Concerning novelty, we think our paper represents a major advancement from previous studies in three ways:

I. ***<u>Systematically engineering the AAA biosynthesis pathway.</u> Although individual or partial combinations of several targets in the AAA biosynthesis pathway have been tested before, to the best of our knowledge, a thorough evaluation of all genes in the AAA biosynthesis pathway has not been reported. Through this, we have identified Pha2 as an important flux controlling step in the production of phenylalanine and its derivatives, and Tyr1 as a limiting step in the production of tyrosine and its derivatives. These are novel engineering targets for the production of aromatic amino acid derived chemicals.***
II. ***<u>Introducing a phosphoketolase (EC 4.1.2.22)-based pathway to divert glycolytic flux towards the formation of E4P.</u> Acting as one of the main bottlenecks in AAA production, the availability of E4P is severely limited in many microorganisms, resulting in a heavy constraint on AAA entry flux, limiting their biosynthesis. The implementation of a phosphoketolase-based pathway with added optimization enabled an almost 100% increase in p-coumaric acid production, compared to the strain (QL158) that lacked***

these carbon rewiring strategies. To emphasize this point, we have added a new figure summarizing the results of flux balance analysis in QL58 (with carbon rewiring strategies) and QL158 (without carbon rewiring strategies) (new Fig. 6c). From these results, it is clear that glycolytic flux has been diverted towards E4P formation and therefore AAA biosynthesis.

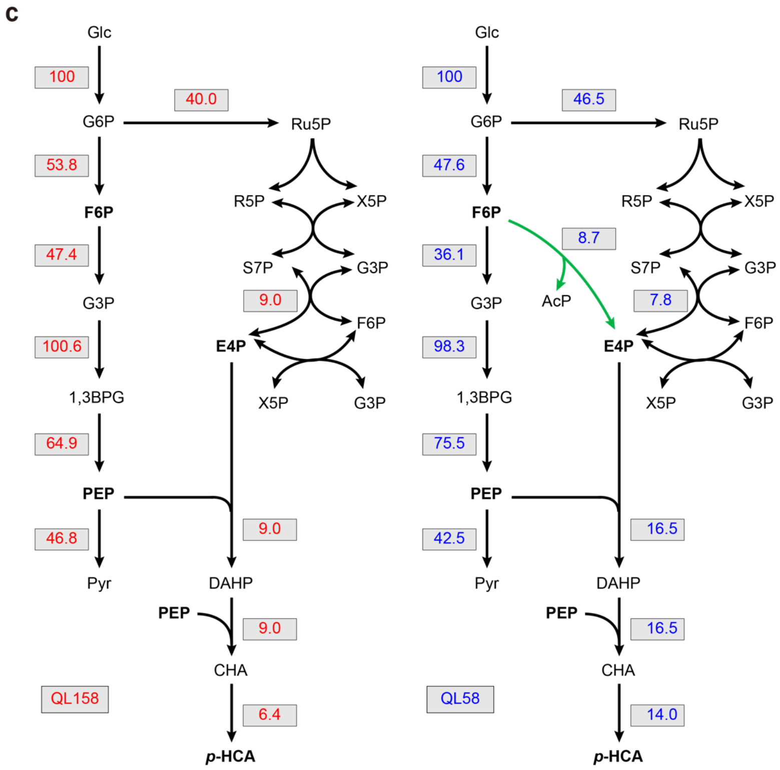

Figure 6c. Predicated metabolic flux distributions via flux balance analysis (FBA) in the engineered medium-level p-HCA producer QL158 (without carbon rewiring strategy) and the high-level p-HCA producer QL58 (with carbon rewiring strategy).

III. ***<u>Optimizing carbon distribution between glycolysis and the AAA biosynthesis pathway by replacing the promoters of several important genes at key nodes between these two pathways, using a promoter library screening approach.</u> There are some strategies which have been previously reported for optimizing precursors PEP and E4P supply (Brochado AR et al., Microb Cell Fact 2010, 9:84; Gold N et al., Microb Cell Fact 2015, 14:73; Curran KA et al., Metab Eng 2013, 15:55-66; Mao J et al., Biotechnol Lett 2017, 39:977-982; Deaner M, Alper HS. Metab Eng 2017, 40:14-22), however, optimizing carbon distribution by replacing the promoters for genes PFK1/2 and PYK1, using a promoter library screening approach has not been performed before. With this strategy, we can directly optimize carbon distribution between glycolysis and AAA biosynthesis, using this irreversible (and optimised) phosphoketolase-based pathway to directly divert carbon flux from glycolysis towards E4P formation and AAA biosynthesis. This is indeed supported by our flux analysis data (new Fig. 6c), which clearly shows carbon redistribution among glycolysis, PPP and the AAA biosynthesis pathway.***

p-Coumaric acid has been a target for several studies, including the one by Rodriguez et al. (10.1016/j.ymben.2015.08.003), which Reviewer 1 mentions. However, the aim of this study is not to focus on p-coumaric acid production but to engineer yeast central carbon metabolism to increase its capability for producing AAAs and their derivates. We would therefore like to emphasize here that we only use p-coumaric acid as a marker to confirm that our strategy has been optimized. Furthermore, p-coumaric acid production based on our engineering strategies showed significant improvements both in titer and yield, compared with previous studies (Table 1), demonstrating that our strategies are valuable for further improving the production of high-value aromatic amino acid derived chemicals.

Another concern is the suitability of this strain as platform strain for the microbial production of flavonoids and other Tyr- and Phe-derived compounds claimed in the very last sentence. This strain has been heavily engineered to provide Tyr and Phe by rerouting the carbon flux to E4P and PEP. However, flavonoids require only 1 mol p-coumaric acid per mol product, but 3 mol malonyl-CoA per mol product. When looking into literature it appears as if colleagues working on flavonoids indeed identified malonyl-CoA as limiting for the synthesis of these valuable compounds, not the p-coumaric acid supply.

Taken together, I think that this manuscript presents some interesting aspects, but is more interesting for readers active in the same field and thus more suitable for a specialized journal.

We thank the reviewer for this comment. We believe that the strategies presented in our paper provide a promising platform for further improvement in the production of many other flavonoids and alkaloids derived from phenylalanine and tyrosine, not necessarily the high-level production of p-coumaric acid only. We have therefore re-written this statement to "Although further optimization may well be required, we are confident the carbon rewiring strategies presented here provide a promising platform for the production of many other flavonoids and alkaloids derived from phenylalanine and tyrosine."
We agree with the reviewer that malonyl-CoA supply can be limiting for flavonoid production, and it is true that the ratio of precursor requirement for p-coumaric acid and malonyl-CoA is 1 to 3, but the mole carbon requirements for both precursors are the same (C9). We have now added new data which extends our pathway from p-coumaric acid to resveratrol (new Supplementary Fig. 19). From these results, it is clear that malonyl-CoA is a limiting precursor for resveratrol production as well. However, p-coumaric acid is also a limiting precursor for resveratrol production. From our data, an increase in p-coumaric acid supply significantly ($p<0.001$) increased resveratrol production by more than 4-fold (strain QL379), compared to strain only with enhanced malonyl-CoA supply (strain QL380). As there is still a lot of p-coumaric acid left in this strain, this would indicate the presence of other limiting factors at this current stage. Since however the aim of this study was not to enhance resveratrol production in yeast, we did not further develop nor optimize for resveratrol production.

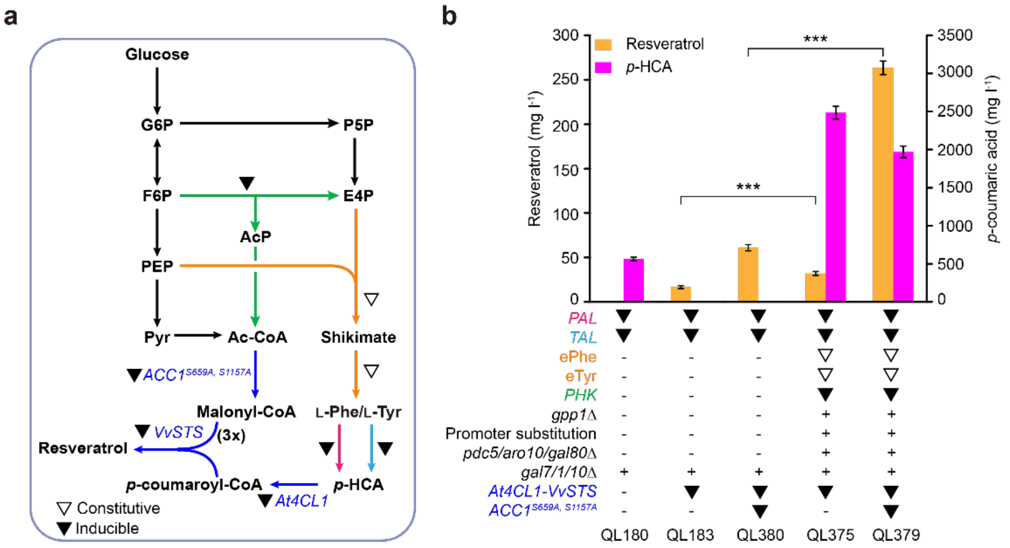

Supplementary Fig. 19. Enabling higher resveratrol production by engineered p-HCA biosynthesis. (a) Schematic illustration of the resveratrol biosynthetic pathway in the context of an enhanced supply of precursor p-HCA. The resveratrol biosynthetic pathway consists of Arabidopsis thaliana 4-coumarate-CoA ligase 1 (At4CL1) and Vitis vinifera stilbene synthase (VvSTS); the deregulated mutant $ACC1^{S659A,\ S1157A}$ was overexpressed to increase the supply of another precursor, malonyl-CoA. Open triangles indicate the use of constitutive strong promoters for controlling gene expression, while filled triangles indicate the use of galactose-inducible promoters. (b) Resveratrol and p-HCA titers obtained with engineered strains. Cells were grown in defined minimal medium with 6 tablets of FeedBeads as the sole carbon source and 1% galactose as the inducer when required. For strains containing a GAL80 deletion, no galactose was supplemented. Cultures were sampled after 96 h of growth and measured for the detection of resveratrol and p-HCA. Statistical analysis was performed using Student's t test (one-tailed; two-sample unequal variance; *$p < 0.05$, **$p < 0.01$, ***$p < 0.001$). All data represent the mean ± SD of biological triplicates.

****

Reviewer 2

This article described the assembly of many metabolic engineering strategies into one yeast strain that brought a production of coumaric acid at a titer of ~3.0 g/L in shake-flask and 12.5 g/L in fed-batch fermentation, which represented the highest level that has ever been achieved in producing the aromatic amino acid pathway derivatives. The manuscript was nicely prepared, including novel interesting interventions such as introducing phosphoketolase to improve E4P availability. Considering that the manuscript included many intermediate strains (summarized in the supplementary information), some of the comparisons need clearer explanations.

1. There was confusion between the strain Q12 in Figure 2 and the strain QL01 in Figure 3. My understanding is that the "the PAL strain labeled in pink" in Figure 3b included all the beneficial manipulations shown in the Figure 2b and 2d, resulting in a titer of ~1.1 g/L. However, QL01 showed production of only 330 mg/L in Figure 3b.

Similarly, QL24 in Figure 3b seemed to overexpress the feedback-insensitive mutant Aro4* and Aro7*. Did the "the PAL strain labeled in pink" already have these two manipulations as shown in QL12 of Figure 2b? Or there were additional copies for Aro4* and Aro7* being inserted into QL24?

We thank the reviewer for this comment and we apologize for the confusion here. To improve clarity here, we have rewritten it so that "PAL" only refers to the pathway converting phenylalanine to p-coumaric acid, which includes the 4 genes AtPAL2, AtC4H, AtATR2 and CYB5. Whereas now we use "ePhe" to refer to modifications (shown in Fig. 2b) that enable the enhancement of phenylalanine production. So, in Figure 3 "PAL" means only the PAL pathway that converts phenylalanine to p-coumaric acid. Therefore, where it was stated "PAL" in Figure 2d this has now been modified to "ePhe" and "PAL", to highlight all the beneficial modifications shown in Figure 2b together with the PAL pathway. This has now been clarified both in the respective figures and in the text.

2. What is the relation between QL290 in Figure 3d (350 mg/L) and QL35 in Figure 4b (~1800 mg/L)? My understanding was that they only differed in the addition of the TAL branch based on the label. However, this enhancement seemed to be dramatic! This was in great contrast when examining QL12, QL18, and QL21 in Figure 2d. The three strains also differed only in the additional genes involved in the Tyr branch, but the increase was only from ~1.1 g/L to 1.6 g/L.
It looked like all the strains in Figure 3 had relatively low titers. Maybe "the PAL strain labeled in pink" (Figure 3) did not include all the manipulations shown in Figure 2?

We thank the reviewer again for this comment. This had been mis-labeled with "PAL" in all aforementioned figures. As mentioned above, the "PAL" strain (labeled in pink) in Figure 3 means only the PAL pathway that converts phenylalanine to p-coumaric acid. This "PAL" background strain is also called "the PAL branch-based strain". "PAL" in Figure 4 and 2d should therefore be "ePhe-PAL" as these strains include all the beneficial modifications shown in Figure 2b. Similarly, "TAL" in Figure 4 should be "eTyr-TAL" as it includes the FjTAL gene and the MtPDH gene shown in Figure 2d. These labels have now been edited with these changes included.

3. Page 8, the second paragraph: it was mentioned, "…which demonstrated more than 40% enhancements upon the same introduction of GPP1 deletion and phosphoketolase pathway (strain QL293 and QL32". Looking at Figure 3d, I found that both strains carried the gpp1 deletion. So the 40% enhancement did not come from gpp1 deletion.

We thank the reviewer for this comment. This again was our mistake. Here it should be strain QL04 and QL32. Introduction of the GPP1 deletion together with the phosphoketolase pathway enhanced the production of p-coumaric acid by 40% (strain QL04 and QL32). If we look at the difference between QL04 and QL24, that is the introduction the PHK pathway in QL24, p-coumaric acid production increased

only by 18% (QL24, Fig. 3b). However, by combining the PHK pathway with the deletion of GPP1, this enabled a ~40% enhancement in p-HCA production, when comparing to QL04 (Fig. 3b and 3d, also shown below). This has now been revised in the text.

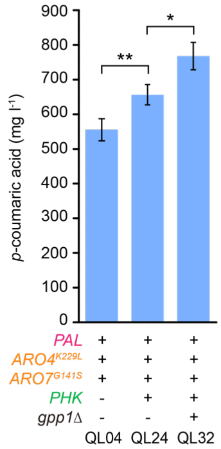

p-HCA production with our engineered strains. Cells were grown in defined minimal medium with 6 tablets of FeedBeads as the sole carbon source. Cultures were sampled after 96 h of growth and measured for the detection of p-HCA. Statistical analysis was performed using Student's t test (one-tailed; two-sample unequal variance; *p < 0.05, **p < 0.01, ***p < 0.001). All data represent the mean ± SD of biological triplicates.

4. Page 9, the bottom two sentences: the authors used QL45, which only had the phophoketolase pathway and Aro4*. What was the starting titer of QL45?

Strain QL45 with the PHK pathway and feedback insensitive Aro4^{K229L} was used to express L-tyrosine hydroxylase, encoded by BvCYP76AD3 variant from Beta vulgaris, and L-DOPA dioxygenase, encoded by MjDOD from the plant Mirabilis jalapa, to convert the colorless L-tyrosine to yellow betaxanthin (Supplementary Fig. 11). This strain therefore does not produce p-coumaric acid. Results for strains with similar backgrounds, however, using the PAL branch or the TAL branch for p-coumaric acid production are shown below.

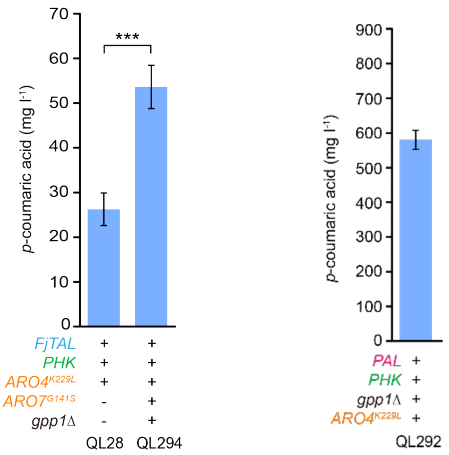

p-HCA production with our engineered strains. Cells were grown in defined minimal medium with 6 tablets of FeedBeads as the sole carbon source. Cultures were sampled after 96 h of

growth for p-HCA detection. Statistical analysis was performed using Student's t test (one-tailed; two-sample unequal variance; *p < 0.05, **p < 0.01, ***p < 0.001). All data represent the mean ± SD of biological triplicates.

The authors used CRISPR technology to introduce the combinatorial promoters for the three target genes and found eight improved variants. How many colonies did they screen? The method section only mentioned 500 colonies per plate.

We screened approximately 300 colonies in total, which we have highlighted in the methods section of the text.
"Transformants were then plated on four SC-URA plates at a density of approximately 500 colonies per plate. Approximately 300 colonies that appeared to have a relatively higher color intensity compared to the rest of the population were selected and inoculated into 1 ml minimal medium and grown for 24 h at 30°C with 200 rpm agitation. The final OD values and color change of the resulting cultures were then compared with that of the background strain QL45 in the first round of screening. Subsequently, the eight most intensely yellow pigmented colonies were isolated for further growth analysis and identified for their promoter integration profile."

5. Page 12, Line #4 from the top: it was mentioned, "overexpression of PHA2 alone being able to increase p-HCA production by 50%". Although this 50% enhancement was demonstrated in Figure S13, it was not consistent with the increase observed from QL05 to QL07 (shown in Figure 2b).

We thank the reviewer for this comment. The backgrounds were different in these two comparisons. In Figure 2b, the overexpression of PHA2 was based on the strain containing the PAL pathway, feedback insensitive Aro4^{K229L} and Aro7^{G141S}, and the shikimate kinase EcAroL. "overexpression of PHA2 (strain QL07, Fig. 2b) led to a significant (p<0.05) increase in p-HCA production by about 23%, compared with strain QL05." But in Figure S18, PHA2 overexpression was in a strain which also contained modifications present within strain QL05, with additional expression of the PHK pathway and a deletion of GPP1, leading to about 50% improvement in p-HCA production. We have now revised this in the text to improve clarity here. "through systematic overexpression of individual genes involved in phenylalanine biosynthesis Pha2 was identified as a bottleneck in p-HCA production (Fig. 2b). This was further proven by overexpression of PHA2 in a strain with further enhanced flux through the shikimic acid pathway, via additional expression of the PHK pathway together with GPP1 deleted, leading to a 50% increase in p-HCA production (Supplementary Fig. 18)."

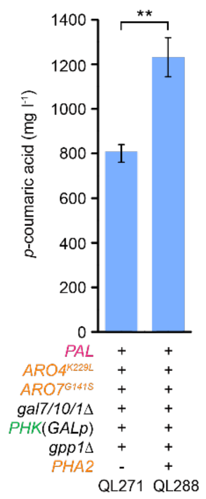

Supplementary Fig. 18. Effect of PHA2 overexpression on p-HCA production. Cells were grown in defined minimal medium with 6 tablets of FeedBeads as the sole carbon source and 1% galactose as the inducer. Cultures were sampled after 96 h of growth for p-HCA detection. Statistical analysis was performed using Student's t test (one-tailed; two-sample unequal variance; *p < 0.05, **p < 0.01, ***p < 0.001). All data represent the mean ± SD of biological triplicates.

6. In Figure S10b, QL48-2 has the promoters designated as "6-5-6". Was this an error since three different promoters "SET3p, CDC24p, and ALD5p" were included in this mutant (Page 10)?
Did SET3p and ILV3p carry similar strengths? How about ALD5p compared to LYS20p?

We thank the reviewer for this comment. The selected promoters have been designated as different numbers, as shown in Supplementary Table 1 and Supplementary Fig. 12. To make this clearer, we have remarked the numbers for the promoters, which were used to replace the promoter of PYK1. So, for strain QL48-2 the promoters should be as "6-5-6*". These labels have now been revised in the text.

Supplementary Table 1. Selected promoters used for fine-tuning the activities of key glycolytic enzymes in Saccharomyces cerevisiae.

No.	Alias	Activity on Glucose[a]	No.	Alias	Activity on Glucose[a]
For *PFK1p/PFK2p* substitution			For *PYK1p* substitution		
Native	*PFK1p/PFK2p*	100%/83%	***Native***	*PYK1p*	100%
1	*ILV3p*	69%	***1****	*RPS11Bp*	42%
2	*ADH3p*	58%	***2****	*RPL23Ap*	30%
3	*VMA6p*	47%	***3****	*YDJ1p*	33%
4	*BAT1p*	36%	***4****	*PRE2p*	19%
5	*CDC24p*	25%	***5****	*RPA12p*	15%
6	*SET3p*	12%	***6****	*ALD5p*	21%
7	*IDP1p*	6%	***7****	*IPP1p*	12%
8	*RAD1p*	5%	***8****	*LYS20p*	82%

9	*PDR3p*	*4%*	***9****	*HXT7p*	*62%*
10	*PDS1p*	*3%*	***10****	*ACS2p*	*37%*

[a] The transcriptional activities of selected promoters are obtained from previous report (Keren, L. et al. Promoters maintain their relative activity levels under different growth conditions. Mol. Syst. Biol. 9, 701 (2013)).

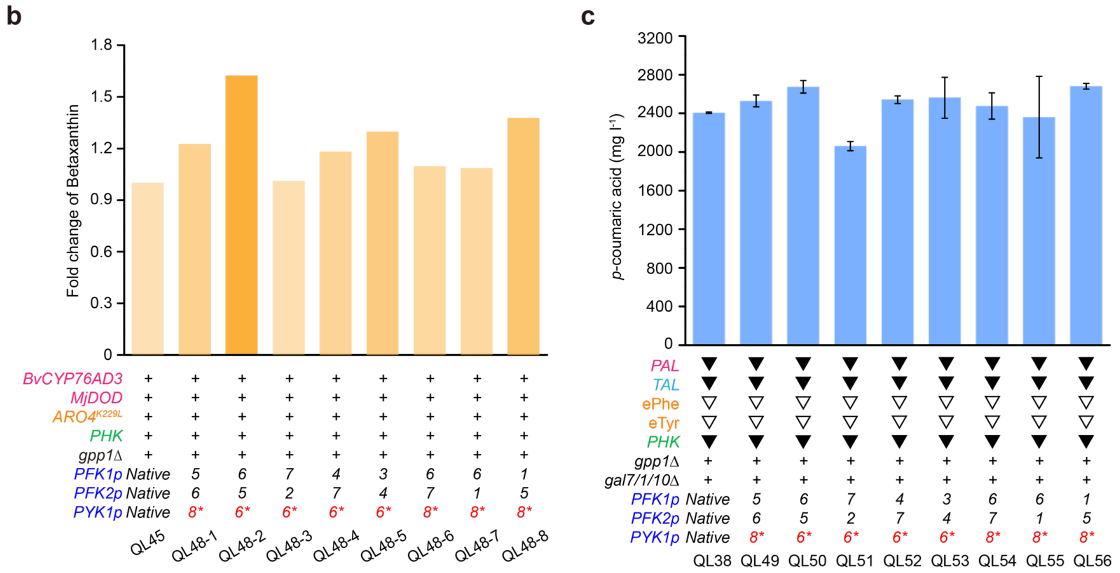

Supplementary Fig. 12. Evaluation of the promoter replaced strains based on betaxanthin screening. (b) Relative accumulation levels (fold change) of betaxanthin in selected promoter-replaced strains compared with strain QL45 as the reference. Values denote the normalized fold change of betaxanthin accumulation per cell mass increase. The color change in each bar reflects the increase in betaxanthin formation. Exact promoters in selected promoter-replaced strains are indicated by their corresponding numbers listed in Supplementary Table 2. (c) Reconstitution of the promoter replacement in the engineered p-HCA producer (QL38) background strain. Open triangles indicate the use of constitutive strong promoters for controlling gene expression, while filled triangles indicate the use of galactose-induced promoters. Cells were grown in defined minimal medium with 6 tablets of FeedBeads as the sole carbon source and 1% galactose as the inducer. All data represent the mean of biological triplicates.

The strength of promoters is depending on the carbon source used during culture cultivation. According to the paper "Keren, L. et al. Mol. Syst. Biol. 9, 701 (2013)", the strength of ILV3p is about 5.9 times stronger than the strength of SET3p on glucose. Similarly, LYS20p is about 3.8 times stronger than ALD5 when comparing on glucose.

7. Page 31, Figure 5c: gal80 deletion was not described in the text.

We have now added this in the text.
"Although deletion of the GAL7/1/10 gene cluster strains were not able to metabolize galactose, these strains still require a low concentration of galactose for induction of the PAL, TAL and PHK pathway genes transcribed by GAL promoters. To avoid the use of galactose, we deleted the GAL80 gene (Fig. 5c), allowing for the induction of galactose regulated genes upon the release of carbon

repression such as by glucose. This approach has been reported to work under similar conditions to ours under glucose-limited fed-batch fermentation conditions without the addition of galactose[38].”
****

Reviewer 3
The manuscript by Liu et al. entitled Rewiring carbon metabolism in yeast for high level production of aromatic chemicals describes metabolic engineering strategies to improve the formation of coumaric acid in Saccharomyces cerevisiae. In addition to approaches that have already published already the study investigate diversion of glycolytic flux through conversion of fructose-6P into E4P and acetyl-P using a phosphoketolase (a subsequent phosphotransacetylase to form acetyl CoA). The combination of old and new genetic modifications led to the construction of strains able to produce 3 g L-1 of coumaric acid, which represent a 50% increase as compare to the best previously published study.

Also impressive, the manuscript is oddly organised, by mixing already demonstrated strategies and new ones, instead of first establishing a reference including all the known beneficial genetic modifications and then evaluate the impact of the new approaches. The makes the manuscript difficult to follow and improvements are reported incrementally from one strain to the next. For instance I do not understand why deletion of PDC5 and ARO10 are only performed in final strains since these modifications were known to impact by-product formation. Additionally the impact of the double deletion is only quantified by increase of coumaric acid concentration. Concentrations of phenylethyl alcohol and its hydroxylated derivative should be presented to the readers.

We thank the reviewer for the constructive comments. We have now added the double deletion of PDC5 and ARO10 into the intermediate strain QL21, which contains all beneficial modifications that were introduced to the AAA biosynthesis pathway (revised Fig. 2d). Based on these new results, the double deletion did not significantly improve p-coumaric acid production. However, implementation of these strategies in the later stage strain, QL38, significantly ($p< 0.05$) improved p-coumaric acid production by 11% (strain QL57, Fig. 5c). From these results we can see that removing by-product production may not be efficient enough to improve target product formation in our early stage strains. However, when the upstream carbon flux was increased in the later stage strains, we did find these modifications to confer a positive effect on final product formation. Furthermore, by quantifying the formation of by-products, we found that phenylethanol, p-hydroxylphenylethanol and the related acids accumulate in strain QL38, while knocking out both ARO10 and PDC5 significantly ($p<0.05$) reduced subsequent by-product formation during p-HCA production (strain QL57, Supplementary Fig. 13). Interestingly, the accumulation of by-products such as phenylethanol and phenylacetate was further increased in strain QL50, when compared to that detected in QL38 (Supplementary Fig. 13), indicating that carbon flux through the AAA biosynthesis pathway had been further increased in QL50. As a result, the double deletions in the promoter substituted strain led to further enhanced p-HCA

production, reaching a titer of 3021.2 mg l-1 in the shake flask cultivation (strain QL58, Fig. 5c), a 26% improvement compared with QL38. These new results therefore, in an indirect way, prove that our carbon rewiring strategies for increasing carbon flux through the AAA biosynthesis pathway was effective.

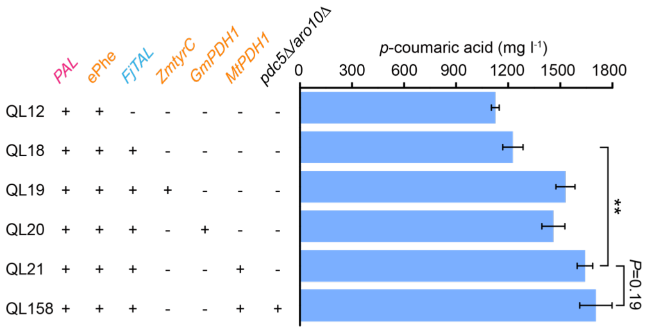

(Revised) Fig. 2d. p-HCA titers obtained with our engineered strains, derived from the combination of PAL and TAL branches and the removal of the AAA degradation pathway. "ePhe" represents the combination of identified upstream beneficial manipulations and "PAL" refers to the PAL branch. Cells were grown in defined minimal medium with 6 tablets of FeedBeads as the sole carbon source, and cultures were sampled after 96 h of growth for p-HCA detection. Statistical analysis was performed using Student's t test (one-tailed; two-sample unequal variance; *p < 0.05, **p < 0.01, ***p < 0.001). All data represent the mean ± SD of biological triplicates.

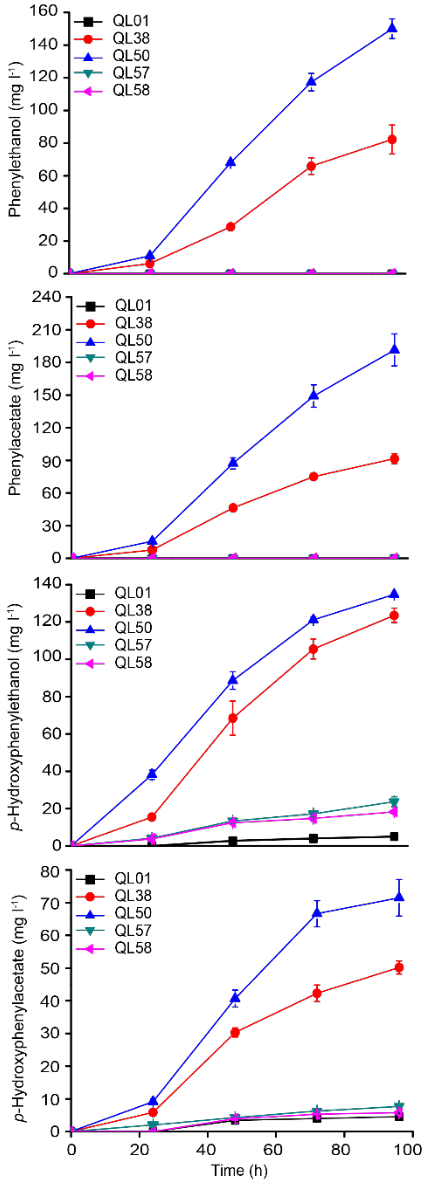

Supplementary Fig. 13. Deletion of PDC5 and ARO10 genes reduce the formation of extracellular fusel alcohols and corresponding organic acids. Production profiles of phenylethanol, phenylacetate, p-hydroxyphenylethanol and p-hydroxyphenylacetate by strains QL01, Ql38, QL50, and QL58 are shown over a 100h time course. Cells were cultivated under shake flask conditions, in defined minimal medium with 6 tablets of FeedBeads as the sole carbon source and 1% galactose as the inducer when required. For strains containing a GAL80 deletion, no galactose was supplemented. Cultures were sampled every 24 h for metabolites detection. All data represent the mean ± SD of biological triplicates.

The deletion of PDC5 and ARO10 also had a significant ($p < 0.05$) effect on cell growth, as shown in the new Supplementary Fig. 4. Consequently, we did not consider including these modifications as part of the background in our reference strain for all subsequent engineering strategies.

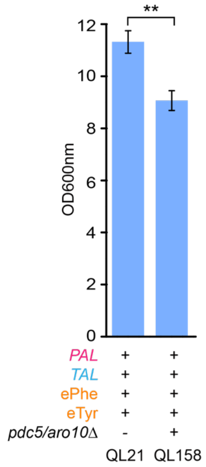

Supplementary Fig. 4. Removal of the AAA degradation pathway reduced cell growth in engineered strains. For shake flask cultivation, cells were grown in defined minimal medium with 6 tablets of FeedBeads as the sole carbon source, and cultures were sampled after 96 h of growth for OD600 measurement. Statistical analysis was performed using Student's t test (one-tailed; two-sample unequal variance; *p < 0.05, **p < 0.01, ***p < 0.001). All data represent the mean ± SD of biological triplicates.

We only tested these modifications in the last strains, where negative impacts on cell growth were less dramatic (new Supplementary Fig. 14).

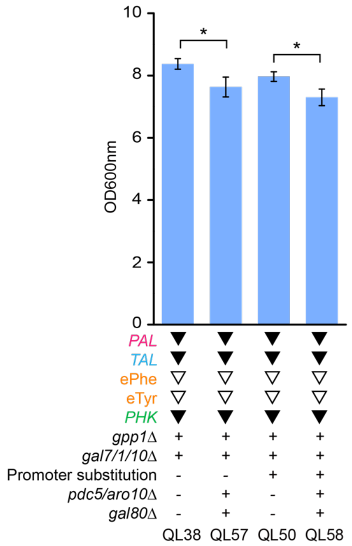

***Supplementary Fig. 14.** Growth profiles of high-level p-HCA production strains with and without removing the AAA degradation pathway. Open triangles indicate the use of constitutive strong promoters to control gene expression, while filled triangles indicate the use of galactose-inducible promoters. For shake flask cultivation, cells were grown in defined minimal medium with 6 tablets of FeedBeads as the sole carbon source and 1% galactose as the inducer when required. For strains containing a GAL80 deletion, no galactose was supplemented. Cultures were sampled after 96 h of growth for OD600 measurement. Statistical analysis was performed using Student's t test (one-tailed; two-sample unequal variance; *p < 0.05, **p < 0.01, ***p < 0.001). All data represent the mean ± SD of biological triplicates.*

Overall the information provided is limited to extracellular concentration of coumaric acid. However most of the speculation in the title, in the motivation of the genetic modifications and in the discussion relate to changes in fluxes. But no data are provided to make such claims. The main remaining question then is what impact on glycolytic and pentose phosphate fluxes all these modifications have? This information would bring the manuscript beyond any other publication showing just incremental improvement.
Providing fluxes redistribution would elaborate on statement made in the introduction: On page 4 line 4 “the authors write “Metabolic flux analysis in yeast has shown that the available carbon flux towards E4P is at least one order of magnitude lower than the flux to PEP, even in a strain that is optimized for initial 5 AAA pathway productivity”. The manuscript should then conclude on the effect of the genetic rewiring of the central carbon metabolism on redistribution of the carbon fluxes.

We thank the reviewer for this constructive comment and based on this we have now added a new figure summarizing the results of a flux balance analysis (new Fig. 6c). To simulate the possible metabolic flux distribution, we used the measured data during glucose-limited fed-batch process from strain QL158 (without carbon rewiring strategies) and strain QL58 (with carbon rewiring strategies). The new results show that the PHK pathway-based carbon rewiring strategies lead to a redistribution of carbon fluxes and increase the available carbon flux towards E4P and aromatic amino acid biosynthesis.

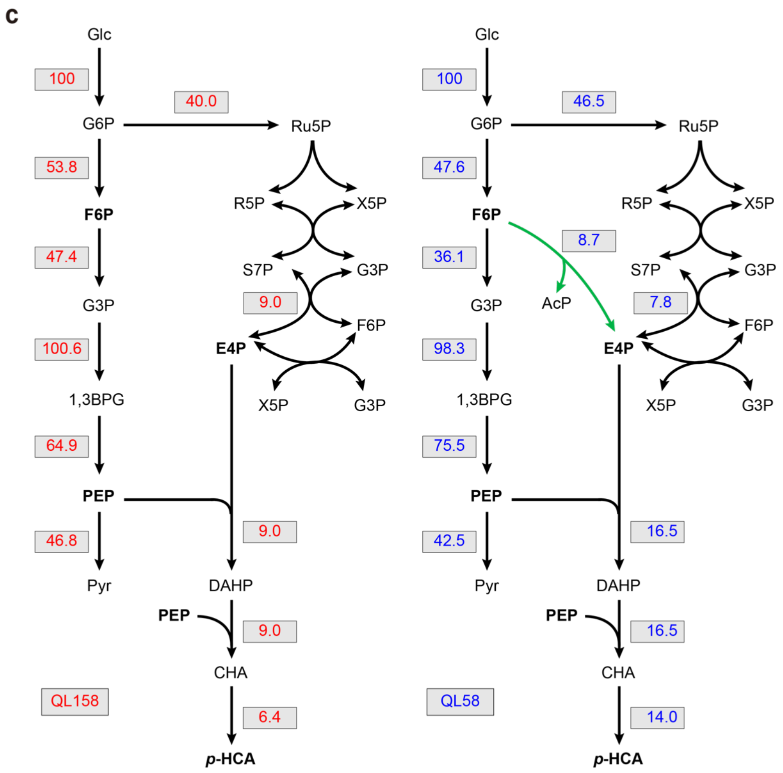

Figure 6c. Predicated metabolic flux distributions via flux balance analysis (FBA) in the engineered medium-level p-HCA producer QL158 (left, without carbon rewiring strategy) and high-level p-HCA producer QL58 (right, with carbon rewiring strategy).

There are several points on which I do not fully agree with the authors:
The authors should comment on the industrial application of strains that express genes under galactose induction. Is this realistic, if not what could then be an alternative. Further the cultivation conditions used for strains requiring galactose induction should be introduced in the results section at least specified that these cultures are not typical batch cultivation.
In a similar manner clearly mentioned that strain QL60 (page 10 last sentence) is grown in fedbatch with 200 g L-1 of glucose as mentioned in the materials and methods section. How do you perform the galactose induction ? This is unclear.

We thank the reviewer for this comment and we apologize for not being clear enough in our description related to this point. From strain QL36 (Fig. 4) we began to use galactose-induced (GAL) promoters to drive the expression of the PAL and TAL pathway respectively, and/or the PHK pathway genes. In order to use galactose only as an inducer and not as a carbon source, we deleted three structural genes GAL1/10/7. A similar principle has been applied to the production of artemisinic acid (Ro, D.K. et al. Nature 440, 940-943 (2006).). In view of galactose regulated genes still being subject to glucose repression, their induction likely occurs after glucose depletion. In our results, all experiments (besides the bioreactor cultivations) were carried out under slow glucose release conditions wherein cells, for most of the time, are under glucose-limited conditions. Therefore, the galactose (1%) used in our shake flask cultivations here could act only as an inducer and not as a carbon source. We have now added galactose induction in the text of Results, Methods and all relevant figure legends.

As galactose is expensive, it would be undesirable to use galactose as an inducer in large scale industrial relevant conditions. Alternatively, GAL80 gene could be deleted to enable the induction of galactose-regulated genes in the absence of a repressing carbon source, such as glucose (Traven, A. et al., EMBO Rep 7, 496-499 (2006).). This has been proven previously for amorpha-4,11-diene production, with results demonstrating that under glucose-limited fed-batch fermentation conditions, without the addition of galactose, strains with gal80 deleted perform similarly to strains that require the addition of galactose to initiate production (Westfall, P.J. et al. Proc Natl Acad Sci U S A 109, E111-118 (2012).). Furthermore, using GAL promoters in a gal80 deletion strain has been demonstrated for high-level artemisinic acid production (Paddon, CJ. et al., Nature, 496, 528-532, (2013).) as well as isoprenoid farnesene production (Meadows, AL. et al. Nature 537, 694-697 (2016).)
In our study, both strain QL58 (haploid) and QL60 (diploid) contained deletions for GAL1/10/7 and GAL80. Therefore, QL60 would have had the same induction mechanism for GAL promoters if glucose catabolite repression was alleviated, such as under glucose-limited conditions. We evaluated QL60 in a bioreactor, in a glucose-limited fed-batch fermentation process, with 200 g l^{-1} glucose as the concentrated feeding carbon source in a feed bottle and with no galactose added as the inducer. Off-site detection of fermentation broth showed the residue glucose concentration during this process to be close to zero (Fig. 6b), confirming that cells

were growing under glucose limited conditions. This has now been additionally clarified in the text.

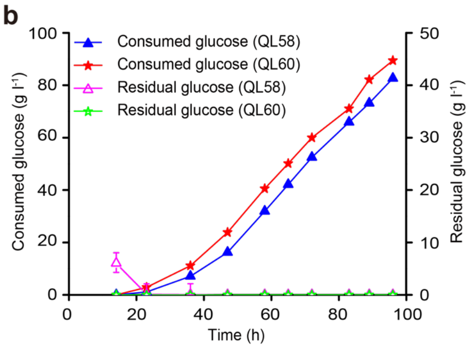

Fig. 6b. Glucose consumption profile (filled symbols) and time course of residual glucose (open symbols) of strain QL58 and its corresponding diploid strain, QL60, during fed-batch fermentation. The data represent the mean ± SD of biological duplicates.

In the first paragraph of the results the authors evaluate the effect of the alleviation of the feedback allosteric loop on DAHP synthase and chorismate mutase. Based on level of coumaric acid only it is concluded that what has been considered in several studies as critical modifications, did not have a significant impact. To be able to conclude this the authors do have to produce ta on pathway intermediates as shikimate, chorismate, phenylethanol and hydroxyl phenylethanol, tryptophol and related acids. Based on these information only an accurate conclusion on the impact of these modifications could be drawn.

We thank the reviewer for this comment. Based on this, we have now added a new supplementary figure summarizing results of these AAA biosynthesis pathway intermediates and by-products (new Supplementary Fig. 2). Based on these results, although p-coumaric acid production was not significantly affected when expressing feedback insensitive DAHP synthase (strain QL02), the concentrations of shikimate, tryptophol, phenylethanol, phenylacetate, p-hydroxyphenylethanol and p-hydroxyphenylacetate were significantly ($p<0.05$) increased, when compared to strain QL01. Expression of feedback insensitive chorismate mutase alone (strain QL03) did not have a significant ($p>0.05$) effect on p-HCA production, nor on pathway intermediates and by-products. When combining both Aro4^{K229L} and Aro7^{G141S} modifications (strain QL04), the pathway intermediate shikimate was further increased, with most by-products also increasing as well, in comparison with strain QL02 (new Supplementary Fig. 2). This was consistent with a previous report on the alleviation of feedback regulation by DAHP synthase and chorismate mutase (Luttik MAH et al. Metab. Eng. 10: 141-153. (2008))). With this new data, we can now clearly see the effect of the alleviation of the feedback allosteric loop on DAHP synthase and chorismate mutase. More importantly, these data support the idea that there were additional bottlenecks existing after expression of Aro4^{K229L} and Aro7^{G141S}, which further supported and improved our paper.

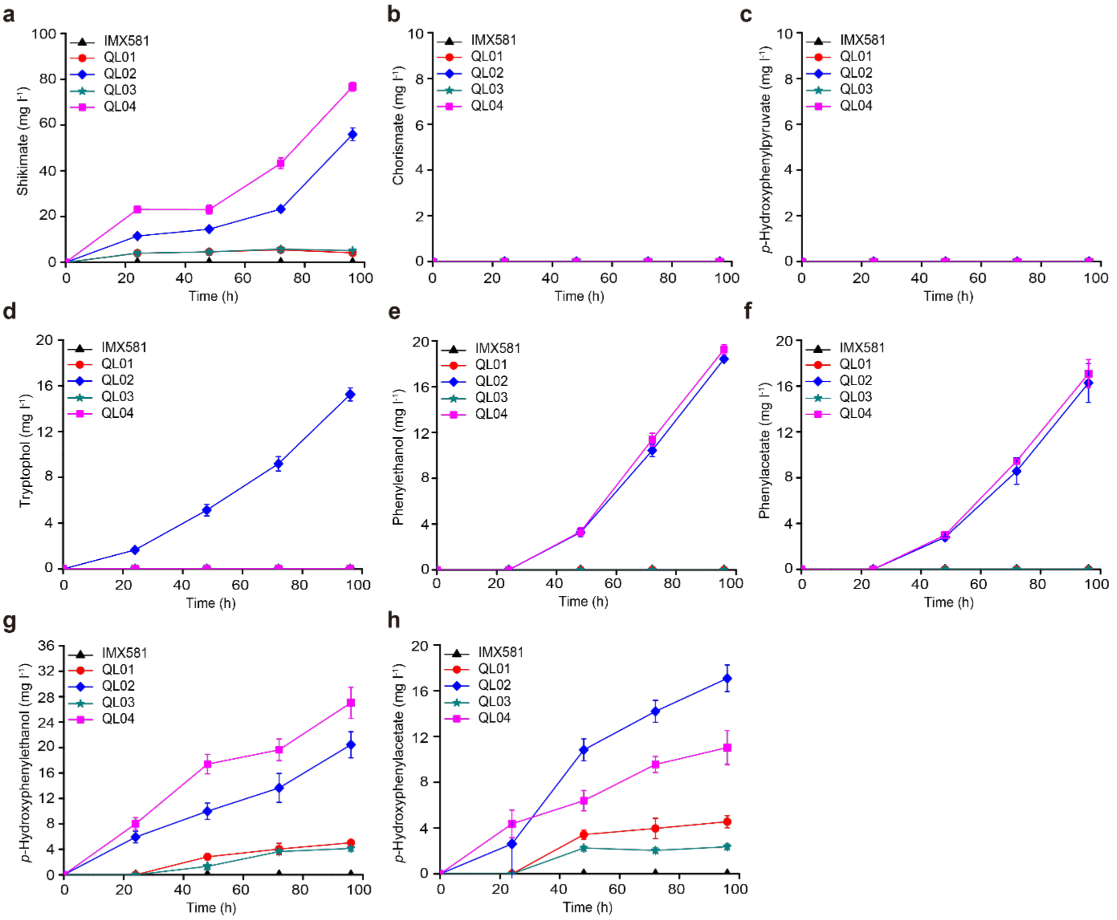

Supplementary Fig. 2. Extracellular concentrations of metabolite intermediates of AAA pathway, fusel alcohols and corresponding organic acids produced by our engineered p-HCA producing strains. Production of (a) Shikimate, (b) Chorismate, (c) p-Hydroxyphenylpyruvate, (d) Tryptophol, (e) Phenylethanol, (f) Phenylacetate, (g) p-Hydroxyphenylethanol and (h) p-Hydroxyphenylacetate by strains QL01, QL02, QL03, QL04 and the wild type strain over a 100h time course. For shake flask cultivation, cells were grown in defined minimal medium with 6 tablets of FeedBeads as the sole carbon source, and cultures were sampled every 24 h for metabolites detection. All data represent the mean ± SD of biological triplicates.

On page 5 “Clearly, the PAL branch was far more efficient compared with the TAL branch, despite intracellular tyrosine concentrations usually reported as being one order of magnitude higher than that of phenylalanine27”, This should be actually thoroughly discussed, several other reports do not support this statement. More accurate quantification of intracellular amino acid concentrations that have not been derived from microtiter plate cultures are publically available.

We thank the reviewer for this comment and apologize overlooking references about this. We have now looked into additional datasets on the quantification of intracellular AAA concentrations in S. cerevisiae. As CEN.PK was used for the strain background in our study, we focused on reports with the same background strain. Intracellular concentrations of Phe and Tyr under different cultivation conditions have been reported as below.

Strain	Metabolite	Intracellular level (µmol gDCW^{-1}) Chemostat (μ = 0.1 h^{-1})	Batch (μ = 0.4 h^{-1})	Reference
CEN.PK 113-7D	Phe	0.47 ± 0.02	1.5 ± 0.1	1
	Tyr	1.41 ± 0.05	1.01 ± 0.06	
CEN.PK 113-7D	Phe	0.87 ± 0.04	0.90 ± 0.15	2
	Tyr	1.25 ± 0.08	0.70 ± 0.09	

1. Canelas, A.B. et al. Quantitative evaluation of intracellular metabolite extraction techniques for yeast metabolomics. Anal Chem 81, 7379-7389 (2009)
2. Canelas, A.B. et al. Integrated multilaboratory systems biology reveals differences in protein metabolism between two reference yeast strains. Nat Commun 1, 145 (2010)

Based on this reported data, it seemed that Phe is generally present at a slightly higher level than Tyr under batch cultivation conditions, while the intracellular level of Tyr is about 1.5-3 times higher than that of Phe, when cells are cultivated under glucose-limited conditions. As we used slow-release glucose Feedbeads or a glucose limited fed-batch process, the intracellular level of Tyr in our strains tested may in fact be a little higher than that of Phe. We have now revised the text to clarify this. "Clearly, the PAL branch was far more efficient compared with the TAL branch, despite intracellular tyrosine concentrations usually being reported as being slightly higher than that of phenylalanine during glucose-limited conditions[28,29], which is similar to our slow-release glucose conditions used here."

The reference 1 and 3 do not report that "flavonoids and alkaloids, are widely used as food and feed additives" could you provide suitable reference for it?

We thank the reviewer for this comment and have now added a new reference on this point. "Kallscheuer, N. et al. Production of plant metabolites with applications in the food industry using engineered microorganisms. Curr. Opin. Biotechnol. 56, 7-17 (2019)"

Reviewers' comments:

Reviewer #1 (Remarks to the Author):

Thank you for the addition of additional material. I think this manuscript can be published.

Reviewer #2 (Remarks to the Author):

The authors have addressed the mistakes and the unclarity in the previous submission. Although a couple of metabolic engineering strategies are somewhat classic, there are novel interventions such as introducing phosphoketolase to improve E4P availability and systematic optimization of the carbon distribution between glycolysis and the aromatic amino acid biosynthesis. Considering the potential of this pathway for producing high-value flavonoids and benzylisoquinoline alkaloids, this work represents a comprehensive engineering endeavor to address the bottlenecks involved in the highly regulated amino acid biosynthesis, resulting in much higher-level production than the titers reported in the existing studies.

Reviewer #3 (Remarks to the Author):

The manuscript Quanli Liu and co-authors entitled “Rewiring carbon metabolism in yeast for high level production of aromatic chemicals” has been revised and still the manuscript is very descriptive enumerating genetic modification after modification without trying to explain the relationship between the constructed genotype and the resulting phenotype. One example is provided by the deletion of the a-keto decarboxylases ARO10 and PDC5, why introduction of these mutations at different stage of the strain construction would result in divergent phenotypes. This point among other has to be discussed. The final strains of the construction scheme harbor a massive amount of genetic alterations that have been introduced through many transformation rounds, I would strongly recommend or demand to analyze the genome sequence of some of the best strains constructed in this study to eliminate the possibility that the phenotype s not linked to other yet uncharacterized events such as ploidy change (chromosome, segment), mutations, … .
This is also critical to be able to evaluate the data from the study with prior art. The discrepancy between the data from Rodriguez et al. (Establishment of a yeast platform strain for production of p-coumaric acid through metabolic engineering of aromatic amino acid biosynthesis. https://doi.org/10.1016/j.ymben.2015.08.003)and those reported in this study, which are one order of magnitude apart.
I would recommend to either move the story about the resveratrol production from discussion to results and describe the associated strain construction and characterization in the materials and methods or either to eliminate it from the manuscript. The data provided are confusing since they are antagonistic with the rest of the manuscript and again are just descriptive (and not linked to any discussion). Not in line with the title of the manuscript

Below we point-by-point address the comments made by each reviewer.

Reviewer #1 (Remarks to the Author):

Thank you for the addition of additional material. I think this manuscript can be published.

We thank the reviewer for their supportive remarks and for helping us improve the manuscript through peer review.

Reviewer #2 (Remarks to the Author):

The authors have addressed the mistakes and the unclarity in the previous submission. Although a couple of metabolic engineering strategies are somewhat classic, there are novel interventions such as introducing phosphoketolase to improve E4P availability and systematic optimization of the carbon distribution between glycolysis and the aromatic amino acid biosynthesis. Considering the potential of this pathway for producing high-value flavonoids and benzylisoquinoline alkaloids, this work represents a comprehensive engineering endeavor to address the bottlenecks involved in the highly regulated amino acid biosynthesis, resulting in much higher-level production than the titers reported in the existing studies.

We thank the reviewer for their positive comments and for helping us improve the manuscript through peer review.

Reviewer #3 (Remarks to the Author):

The manuscript Quanli Liu and co-authors entitled "Rewiring carbon metabolism in yeast for high level production of aromatic chemicals" has been revised and still the manuscript is very descriptive enumerating genetic modification after modification without trying to explain the relationship between the constructed genotype and the resulting phenotype. One example is provided by the deletion of the a-keto decarboxylases ARO10 and PDC5, why introduction of these mutations at different stage of the strain construction would result in divergent phenotypes. This point among other has to be discussed.

We thank the reviewer for this constructive comment. Deletion of *ARO10* and *PDC5* in the early stage strain QL21 (resulting into QL158) did not significantly increase *p*-coumaric acid production (Fig. 2d). However, implementation of the double deletion in the later stage strain QL38 significantly improved *p*-coumaric acid production by 11% (strain QL57, Fig. 5c). We think that it is exactly the "different stage of the strain construction" (strain backgrounds) can be attributed to this divergence. From these results we can see when the upstream carbon flux was increased in the later stage strains, we did find these modifications to confer a positive effect on final product formation. On the other hand, we have to notice that the dynamic control strategies implemented in the later stage strains had also positive effects on *p*-coumaric acid production. In strain QL21 (and QL158) both the PAL and TAL branch genes were under the control of constitutive promoters, while in strain QL36 dynamic control via inducible promoters were applied to control the expression of the PAL and TAL branches, which significantly increased *p*-coumaric acid production by 10% (Figure 4c). These results implied that channeling AAAs to the production of *p*-coumaric acid could be a limiting factor, as *ARO10* and *PDC5* deletions in QL158

did not redirect carbon flux towards *p*-HCA biosynthesis. With optimized downstream flux towards product formation, inducible expression of the PHK pathway (QL38) led to further increase in *p*-coumaric acid production by 33%. Furthermore, double deletion of *ARO10* and *PDC5* in this background (QL38) resulted in an additional 11% increase of *p*-HCA production (QL57, Fig. 5c). Therefore, the divergent phenotypes were due to different strain backgrounds. Specifically, in the later stage strains there were not only increased upstream carbon flux, but also better-optimized carbon flux towards the product (*p*-coumaric acid) formation via dynamic control. Therefore, the double deletions in the late stage strains resulted into significant improvements in *p*-coumaric acid production. We have amended the Discussion section to address these points.

The final strains of the construction scheme harbor a massive amount of genetic alterations that have been introduced through many transformation rounds, I would strongly recommend or demand to analyze the genome sequence of some of the best strains constructed in this study to eliminate the possibility that the phenotype s not linked to other yet uncharacterized events such as ploidy change (chromosome, segment), mutations, ⋯ .

We thank the reviewer for this comment. This is a very good point, but we always evaluated (at least) three independent biological colonies for shake-flask fermentation and the evaluation of *p*-HCA production, to ensure the reliability and reproducibility of presented data. It is very unlikely that unspecified mutations could confer the phenotypes observed. Also, this is not a common practice in metabolic engineering, such as engineering of yeast for production of farnesene, artimisinic acid and canabinoids (Meadows et al., Nature 2016, 537:694-697; Paddon et al., Nature 2013, 496:528-532; Luo et al., Nature 2019, 567:123-126). Therefore, we do not think that genome sequence of engineered strains would yield any meaningful outcomes. However, if the editor disagrees with us on this issue, we can of course have the strain sequenced.

This is also critical to be able to evaluate the data from the study with prior art. The discrepancy between the data from Rodriguez et al. (Establishment of a yeast platform strain for production of p-coumaric acid through metabolic engineering of aromatic amino acid biosynthesis. https://doi.org/10.1016/j.ymben.2015.08.003)and those reported in this study, which are one order of magnitude apart.

We thank the reviewer again for this comment. We have discussed the discrepancy of utilizing TAL branch for *p*-HCA production between the previous report by Rodriguez et al. and our study (page 13 L4-13). This huge difference we think was due to different medium and cultivation conditions used. We used minimal medium under batch conditions, while Rodriguez et al. used commercial fed-batch complex medium, which is not available anymore. There were also other differences between these two studies, i.e. components of gene constructs like promoters, terminators, and integration sites, which may also explain the different results obtained. We have added this in the Discussion part. Also, the aim of this study is not to focus on *p*-coumaric acid production but to engineer yeast central carbon metabolism to increase its capability for producing AAAs and their derivates. We would therefore like to emphasize here that we only use *p*-coumaric acid as a marker to confirm that our strategy has been optimized.

I would recommend to either move the story about the resveratrol production from discussion to results and describe the associated strain construction and characterization in the materials and

methods or either to eliminate it from the manuscript. The data provided are confusing since they are antagonistic with the rest of the manuscript and again are just descriptive (and not linked to any discussion). Not in line with the title of the manuscript

We thank the reviewer for this comment. Strain construction and characterization for resveratrol production were already described in the Materials and Methods section (page 20 and 22). In the results part we used *p*-coumaric acid as a marker to demonstrate that carbon rewiring strategies in yeast were effective to increase its capability for producing AAAs and their derivates. To discuss and showcase the utility of our optimized strains in the production of other value-added aromatic compounds, resveratrol biosynthetic pathway was engineered and evaluated in our engineered strains (Supplementary Fig. 19). Our data of resveratrol production on one hand showed that the strategies we employed were effective to increase another aromatic compound - resveratrol production. On the other hand, high-level accumulation of *p*-HCA in strains QL375 and QL379 further demonstrated that our engineering strategies were efficient in rewiring cellular metabolism for AAAs biosynthesis. In addition, the production of resveratrol in our study is comparable with the highest level reported for production of this compound in shake-flask cultivation of yeast cells. We therefore think this result is compatible with the rest of our manuscript and also in line with the title of manuscript.

REVIEWERS' COMMENTS:

[Editor: Reviewer #3 states his/her comment in Remark to Editor section. This reviewer thinks whole genome sequencing of engineered strains should become the norm. However, (s)he is fine with this version and accept it as such.]

Below we point-by-point address the comments made by each reviewer.

REVIEWERS' COMMENTS:

Reviewer #3 states his/her comment in Remark to Editor section. This reviewer thinks whole genome sequencing of engineered strains should become the norm. However, (s)he is fine with this version and accept it as such.

We thank the reviewer for their supportive remarks and for helping us improve the manuscript through peer review.
